# One-Pot Synthesis of (Z)-β-Halovinyl Ketones via the Cascade of Sonogashira Coupling and Hydrohalogenation

**DOI:** 10.3389/fchem.2020.621545

**Published:** 2021-04-22

**Authors:** Fa-Jie Chen, Zhenguo Hua, Jianhui Chen, Jiajia Chen, Daesung Lee, Yuanzhi Xia

**Affiliations:** ^1^College of Chemistry and Materials Engineering, Wenzhou University, Wenzhou, China; ^2^Department of Chemistry, University of Illinois at Chicago, Chicago, IL, United States

**Keywords:** Sonogashira coupling, one-pot synthesis, β-halovinyl ketone, hydrohalogenation, ynones

## Abstract

Herein, we report an efficient method for the synthesis of (Z)-β-halovinyl ketones through a one-pot Sonogashira coupling and hydrohalogenation reaction promoted by palladium-copper catalyst and Brønsted acid. The ynone intermediates are generated *in situ* from readily available acid chlorides and terminal alkynes at room temperature, which are directly converted to (Z)-β-halovinyl ketones by treating with triflic acid. This method avoids the use of an external halogen source and features broad substrate scope, high yield, and good to excellent stereoselectivity.

## Introduction

The palladium and copper-catalyzed cross-coupling reaction of terminal alkynes with aryl or vinyl halides, also known as Sonogashira reaction, has emerged as a powerful method for the synthesis of substituted alkynes in recent decades (Sonogashira et al., 1975; Sonogashira, 2002). The scope of this reaction has been extended to acid halides, which reacts with terminal alkynes smoothly at room temperature to provide ynones in good yield (Tohda et al., 1977; Eckhardt and Fu, 2003). The electron-deficient ynones are good Michael acceptors and react with various nucleophiles to form vinyl ketones (such as enaminone and β-ketoenolether) and heterocycles (such as pyrimidine) (Karpov and Müller, 2003a,b). Ynones are also used as useful precursors for the synthesis of β-halovinyl ketones (Pohland and Benson, 1966; Goossen et al., 2009).

**Scheme 1:**
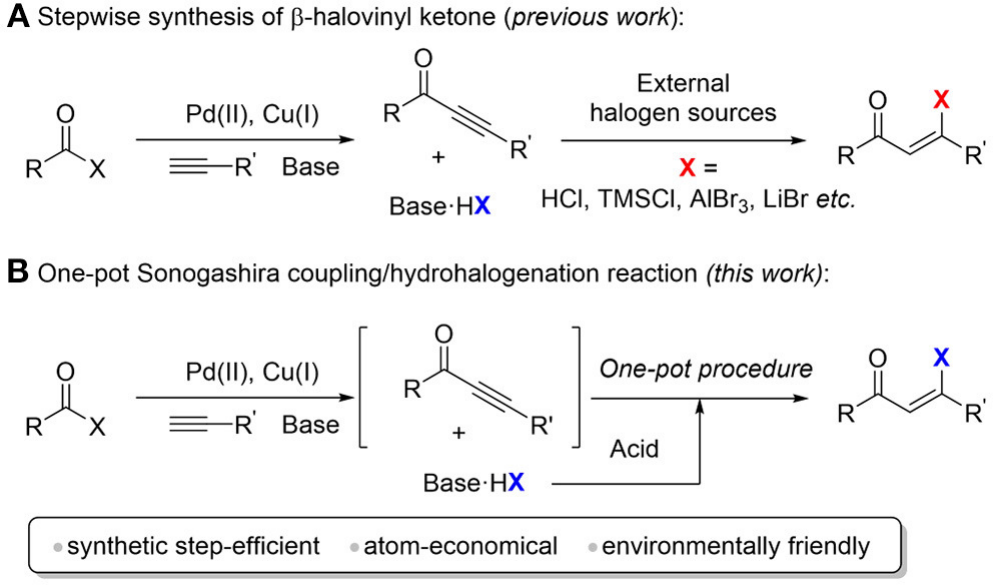
Synthesis of β-halovinyl ketones. **(A)** Stepwise synthesis of β-halovinyl ketone (*previous work*). **(B)** One-pot sonogashira coupling/hydrohalogenation reaction *(this work)*.

β-Halovinyl ketones are important building blocks in organic synthesis and biochemical processes, and they have been widely used for the constructions of heterocycles such as chromenones, furans, pyrazole, and pyridines (Kim et al., 2012, 2017; Kim and Oh, 2014, 2015, 2017, 2020; Koo et al., 2019a). The methods to form β-halovinyl ketones from ynones generally involve regioselective hydrohalogenation of electron-deficient carbon-carbon triple bond. In these cases, a variety of halogen sources have been employed, including HCl, SnCl_4_, AlBr_3_, TMSCl, and LiBr (Scheme 1A) (Kundu and Chaudhuri, 1991; Shchukin and Vasilyev, 2008; Yang et al., 2011; Semenova et al., 2013; Yan et al., 2015; Zeng et al., 2017; Zhang et al., 2017). However, those methods suffer from relatively low stereoselectivity, which limits their synthetic applications. Recently, Xu and co-workers reported an atom-economical method for the regio- and stereoselective hydrohalogenation of ynones and ynamides using DMPU/HX (X = Br or Cl) reagents. Paixão and co-workers reported the regioselective synthesis of (Z)-β-halo α,β-unsaturated carbonyl systems via combination of halotrimethylsilane and tetrafluoroboric acid (da Silva et al., 2019).

Ynones used in these hydrohalogenation methods are usually not commercially available. One of the common ways to prepare the precursor ynones is the Sonogashira cross-coupling reaction as mentioned above (Kokubo et al., 1996; Hua et al., 2005; Kashiwabara et al., 2005, 2008; Iwai et al., 2009, 2012; Kashiwabara and Tanaka, 2011). This hydrohalogenation strategy for β-halovinyl ketone synthesis requires a multi-step synthetic procedure (Scheme 1A). Moreover, in the starting material preparation step, at least one equivalent of chemical waste hydrohalide salt (for example, triethylamine hydrochloride) will be generated, which is commonly removed in the workup procedure. From the viewpoint of atom-economy and green chemistry, we envisioned that the chemical waste hydrohalide salt can be reused as the halogen source for the sequential hydrohalogenation to form β-halovinyl ketones by the one-pot treatment of strong Brønsted acid, thus avoiding the use of an external halogen source. Compared to the stepwise methods, this strategy is more step- and atom-economical and environment friendly.

Similar strategies have been developing relying on transition-metal (rhodium and iridium catalyst) (Kokubo et al., 1996; Hua et al., 2005; Kashiwabara et al., 2005, 2008; Goossen et al., 2009; Iwai et al., 2009, 2012; Kashiwabara and Tanaka, 2011), or Lewis acid (Price and Pappalardo, 1950; Benson and Pohland, 1964; Martens et al., 1975; Zhou et al., 2006; Wang et al., 2010; Hosseini-Sarvari and Mardaneh, 2011; Gandeepan et al., 2012; Koo et al., 2019b). However, these developed methods usually require relatively harsh reaction conditions, such as elevated temperature, long reaction time, use of expensive ligands or catalysts. To the best of our knowledge, the efficient synthesis of β-halovinyl ketones at room temperature through the cascade of the Sonogashira coupling and hydrohalogenation has not been reported.

Herein, we developed a one-pot synthesis of (Z)-β-halovinyl ketone via a Sonogashira coupling and hydrohalogenation sequence at room temperature (Scheme 1B). The ynone intermediates are generated *in situ* from the palladium and copper catalyzed cross coupling of acid halides and terminal alkynes, which are hydrohalogenated to afford (*Z*)-β-halovinyl ketone using side product hydrohalide salt as a halogen source. This method shows good stereoselectivity, high yield, and broad substrate scope.

## Results and Discussion

We commenced our study with the synthesis of β-chlorovinyl ketone **3a** through a cross-coupling reaction of phenylacetylene **1** with benzoyl chloride **2** under the catalysis of PdCl_2_(PPh_3_)_3_ and CuI followed by Brønsted acid treatment (Table 1). After systematic optimization of the reaction conditions, we found that the best reaction conditions: 1.0 equivalent of phenylacetylene reacts with 1.3 equivalent of benzoyl chloride in the presence of 2 mol% of PdCl_2_(PPh_3_)_2_, 4 mol% of CuI, and 1.2 equivalent of triethylamine in 1,2-dichloroethane (0.4 M) at room temperature for 10 min, then treat the reaction mixture with 1.5 equivalent of triflic acid for 4 h at room temperature. The stereoselectivity for this transformation is up to 91/9 (*Z/E*) and (*Z*)-β-chlorovinyl ketone **3a** was obtained in 87% yield (Table 1, entry 14). When triflic acid was replaced with weaker Brønsted acids, such as trifluoroacetic acid (entry 4), acetic acid (entry 5), and benzoic acid (entry 6), chlorovinyl ketone **3a** was not generated. This result indicates the crucial role of triflic acid to react with triethylamine hydrochloride to release hydrochloride acid, which acts as the chloride source for the following hydrochlorination step. Other solvents such as ethanol (entry 9) and acetonitrile (entry 10) are not effective for this transformation. Extra co-solvent for the hydrohalogenation step is not necessary. Shorten the reaction time of Sonogashira coupling step to 2 min results in a decrement of the yield (65%) and stereoselectivity (86/14, *Z/E*).

**Table 1:**
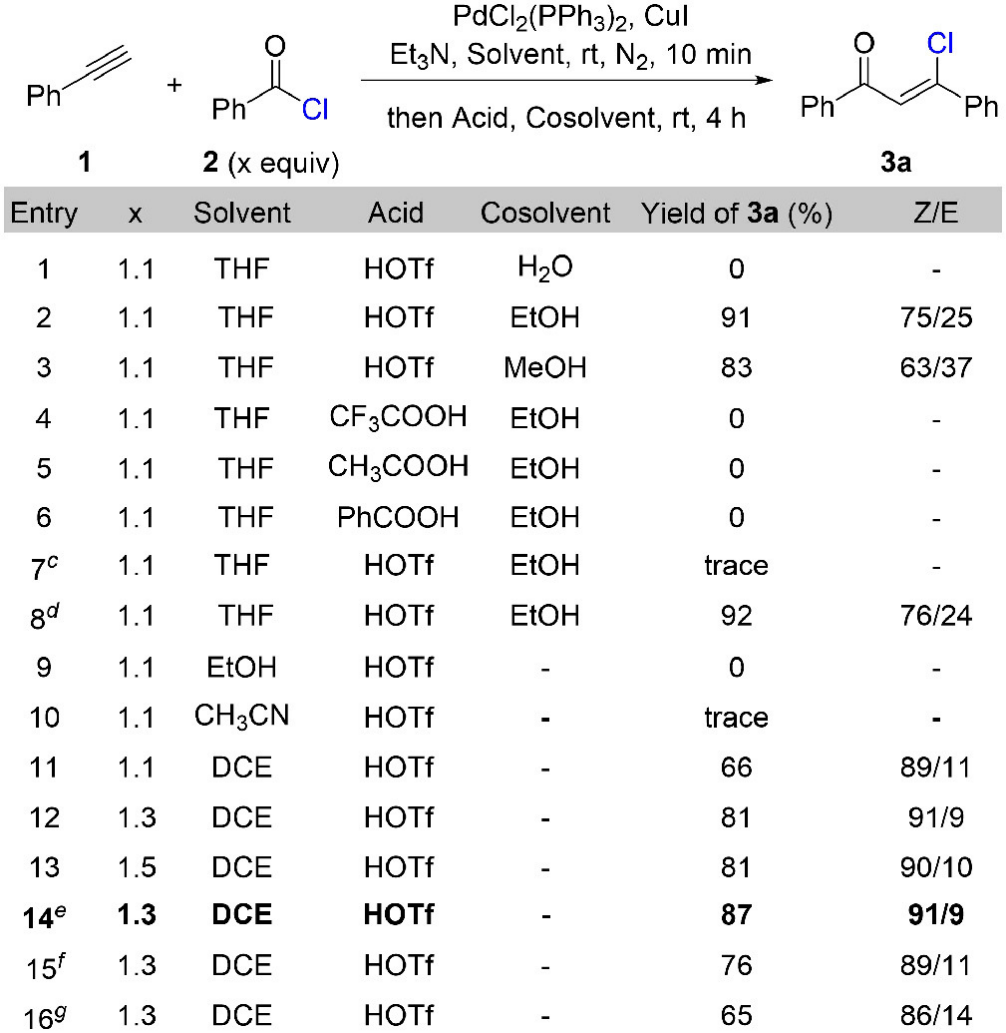
Optimization of reaction conditions^*a*^^,^^*b*^.

a *Reaction conditions: **1** (0.2 mmol), **2** (x equiv), PdCl_2_(PPh_3_)_2_ (2 mol%), CuI (4 mol%), Et_3_N (1.2 equiv) in solvent (1.0 mL) at rt under N_2_ for 10 min, then acid (1.5 equiv) in cosolvent at rt for 4 h unless otherwise noted. ^b^Isolated yield. ^c^0.5 equiv of HOTf. ^d^2 equiv of HOTf. ^e^0.5 mL of DCE. ^f^1.5 mL of DCE. ^g^The reaction time of the first step is 2 min*.

With the optimized reaction conditions in hand, we started to explore the substrate scope of this protocol. As showed in Table 2, a wide range of terminal alkynes were suitable in this transformation. Both electron-withdrawing groups, such as Br (**3e**), Cl (**3b**, **3c**), F (**3d**) and electron-donating groups, such as Me (**3f**), OMe (**3g**) on the aromatic rings are well-tolerated, giving the (*Z*)-β-chlorovinyl ketone in good to high yield as well as good stereoselectivity. An alkyne bearing a thiophene moiety is also a good coupling partner, giving β-heterocycle-substituted unsaturated ketone **3i** in 75% yield with a 92/8 ratio of *Z/E* isomers. The *Z/E* ratio of the product can be further increased up to 99/1 when sterically hindered alkynes were used (**3l**). The low yields were obtained for hydroxyl group-containing products (**3h** and **3j**). It is likely because the hydroxyl group is acid sensitive. Replacement with a less sterically hindered linear 1-hexyne results in a dramatic decrease of the stereoselectivity of the ketone **3k** to 41/59 (*Z/E*), indicating a bulky substituent at the alkyne moiety is important for high stereoselectivity. Trimethylsilyl-containing chlorovinyl ketone **3m** was obtained in 63% yield and 89/11 (*Z/E*) stereoselectivity.

**Table 2:**
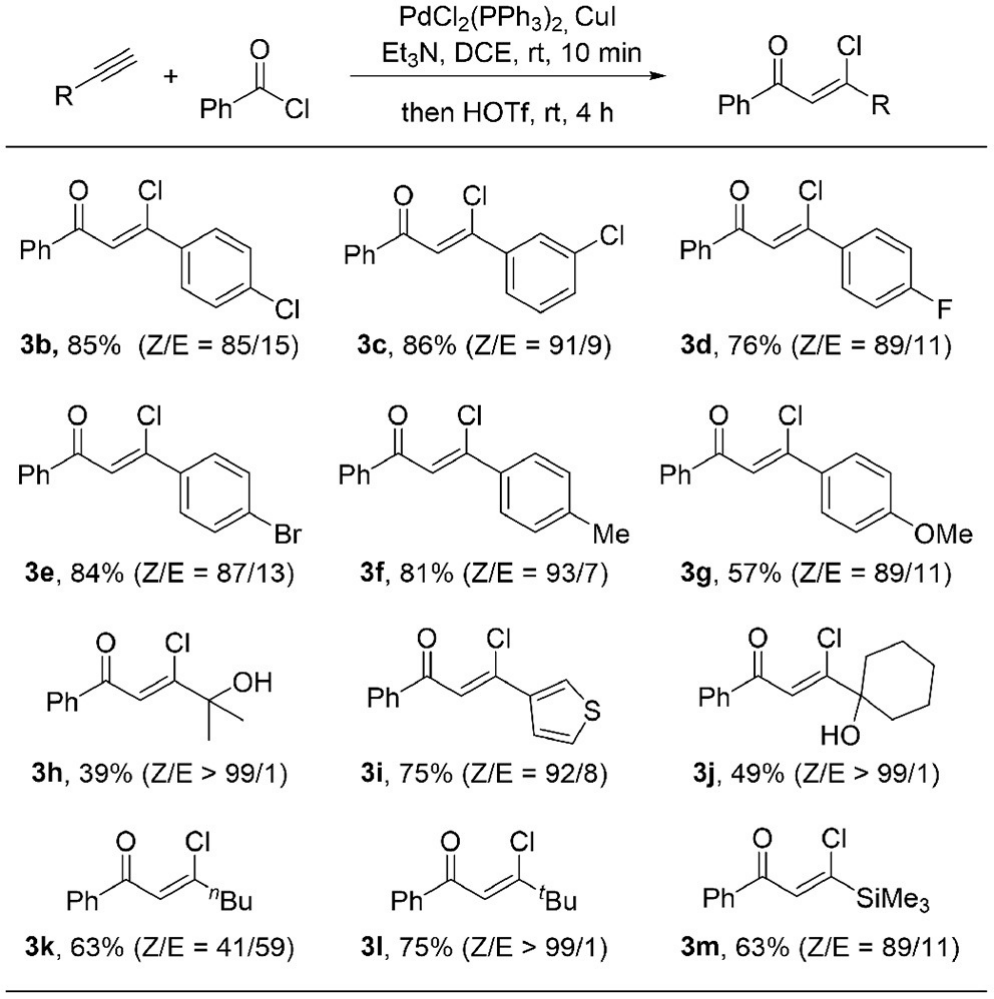
Substrate scope of alkynes^*a*^^,^^*b*^.

a *Reaction conditions: alkyne (0.2 mmol), acyl chloride (0.26 mmol), PdCl_2_(PPh_3_)_2_ (2 mol%), CuI (4 mol%), Et_3_N (1.2 equiv) in DCE (0.5 mL) at rt under N_2_ for 10 min, then HOTf (1.5 equiv) at rt for 4 h. ^b^Isolated yield*.

Encouraged by the broad substrate scope of alkynes, we further examined the generality of the acyl chloride counterpart under standard reaction conditions (Table 3). A variety of benzoic acid chlorides substituted with either electron-donating groups (such as Me, OMe) or electron-withdrawing groups (such as F, Cl, Br) react with phenylacetylene and 1-hexyne smoothly, giving β-chlorovinyl ketone in good to excellent yield and high stereoselectivity. Inclusion of a strong electron-withdrawing CN group results **4l** in only 27% yield, while the **4m** could be formed in 60% yield when this was replaced by CF_3_. The *Z/E* ratio of the product is up to 99/1 in most cases. Ketone **4n** was obtained in 82% yield from the reaction of aliphatic acid chloride. Thiophene containing product **4o** was also obtained in 77% yield and excellent *Z/E* selectivity (>99/1).

**Table 3:**
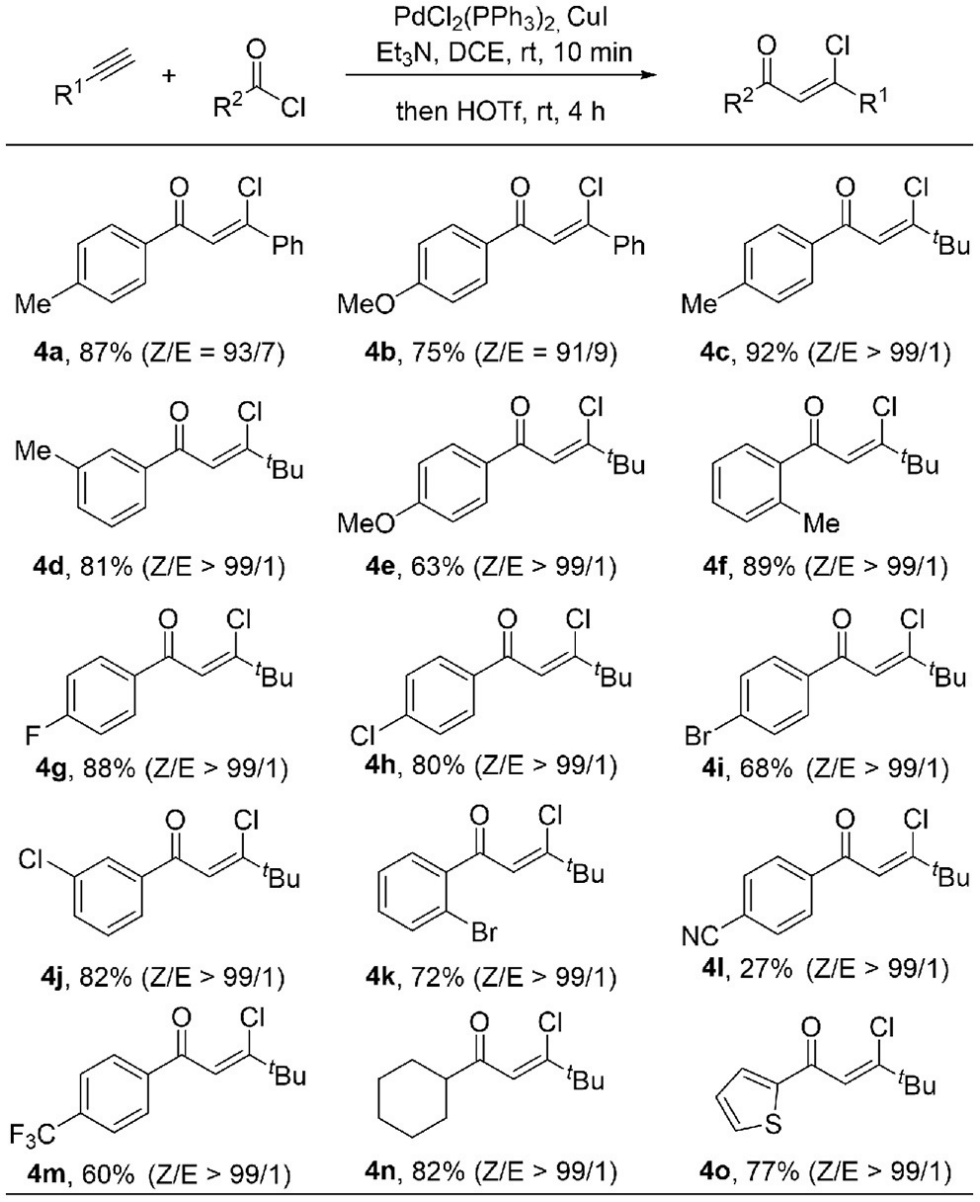
Substrate scope of acyl chloride^*a*^^,^^*b*^.

a *Reaction conditions: alkyne (0.2 mmol). acyl chloride (0.26 mmol), PdCl_2_(PPh_3_)_2_ (2 mol%), CuI (4 mol%), Et_3_N (1.2 equiv) in DCE (0.5 mL) at rt under N_2_ for 10 min, then HOTf (1.5 equiv) at rt for 4 h. ^b^Isolated yield*.

**Scheme 2:**
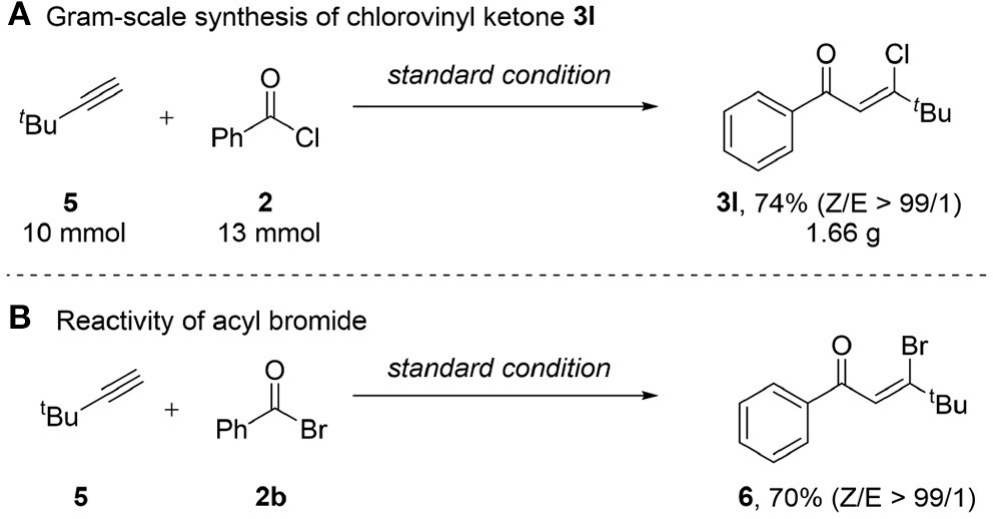
Synthetic application of the protocol. **(A)** Gram-scale synthesis of chlorovinyl ketone 3I. **(B)** Reactivity of acyl bromide.

To demonstrate the application of this method in organic synthesis, a gram scale experiment was carried out (Scheme 2A). We were delighted to find that the reaction of *tert*-butylacetylene **5** (10 mmol) with benzoic acid chloride **2** gave ketone **3l** in gram-scale with high yield and stereoselectivity. As acid chlorides have been proved to be efficient for this transformation, we then turned our attention to the reactivity of acyl bromide with benzoic acid bromide (Scheme 2B), which gave rise to the β-bromovinyl ketone **6** in satisfactory yield (70%) with excellent *Z/E* ratio (99/1). However, reaction of **5** with benzoic acid iodide resulted a complex mixture.

**Scheme 3:**
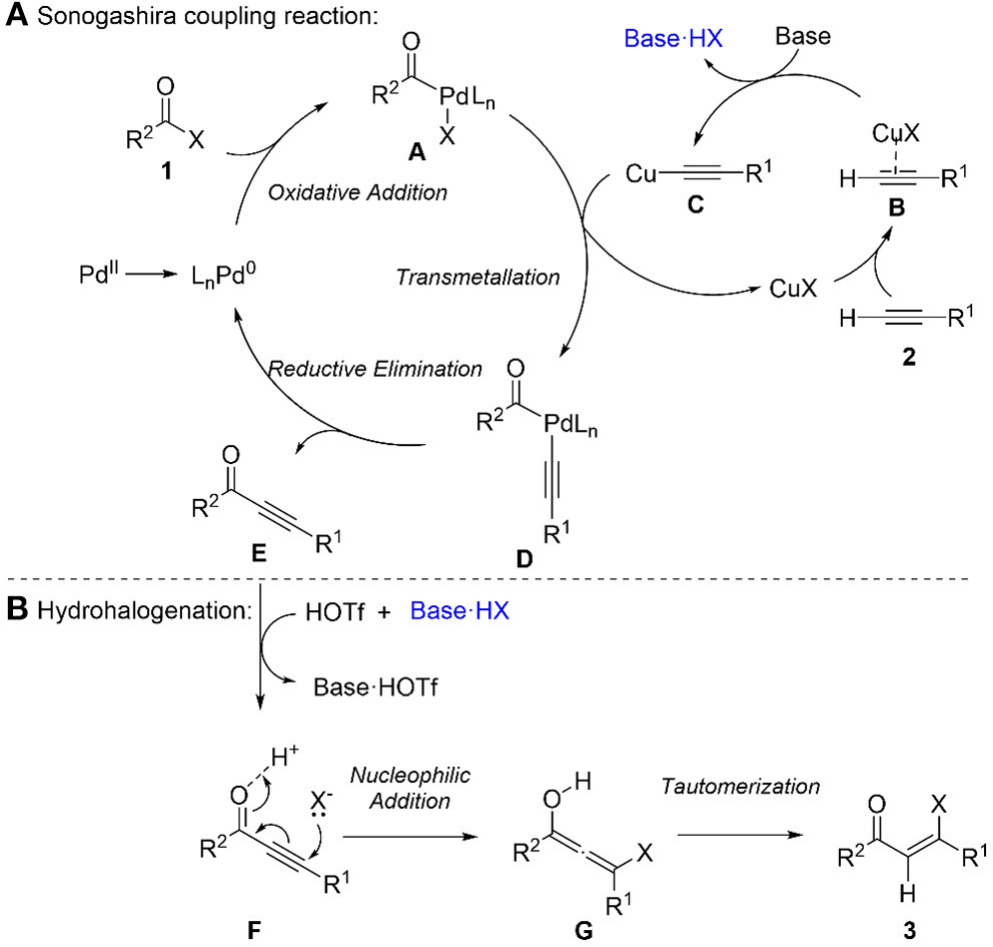
Proposed mechanism. **(A)** sonogashira coupling reaction. **(B)** hydrohalogenation.

Based on the experimental results and precedents (Chinchilla and Nájera, 2007), a plausible mechanism for this sequential Sonogashira reaction/hydrohalogenation reaction was proposed (Scheme 3). Firstly, palladium precatalyst PdCl_2_(PPh_3_)_2_ forms a reactive Pd(0) species (Amatore and Jutand, 2000), which undergoes oxidative addition with acyl chloride to produce intermediate **A**. Secondly, the transmetallation of intermediate **A** with copper acetylide **C**, which is formed from terminal alkyne via complexation and deprotonation, gives intermediate **D**. Sequently, reductive elimination of **D** affords ynone and regenerated Pd(0) species. For the hydrohalogenation step, halogen source HX (such as HCl) is generated from the reaction of triflic acid with triethylammonium chloride formed in Sonogashira coupling step. The Michael addition of the halide source to the ynone **E** generates an allenyl intermediate **G**, which tautomerizes to the final product β-halovinyl ketone **3**.

## Conclusion

We have developed an efficient method for the synthesis of (*Z*)-β-halovinyl ketones from readily available terminal alkynes and acid chlorides through sequential Sonogashira coupling/hydrohalogenation reaction. This method features simple operations, high yield, and excellent stereoselectivity in most cases. It can be easily scaled up to a gram scale. The broad substrate scopes of both alkyne and acyl chloride show its potential application in organic synthesis.

## Experimental Section

### General Information

Unless otherwise noted, all chemicals were purchased from J&K, Energy-Chemical or Tansoole, and used as received. All reactions were carried out using oven-dried glassware and magnetic stirring under N_2_. The product was monitored and tracked by TLC (iodine, potassium permanganate, and other color reagents will be used if necessary). The product was extracted and filtered with 100–200 mesh silica gel and separated and purified with chromatography silica gel column or chromatography silica gel plate (specification of silica gel is 300–400 mesh silica gel). ^1^H NMR spectrum and ^13^C NMR spectrum were determined by Bruker-400 instrument or Bruker-500 instrument with TMS as internal standard and deuterium chloroform as solvent. ^1^H NMR chemical shifts were referenced to tetramethylsilane signal (0 ppm), ^13^C NMR chemical shifts were referenced to the solvent resonance (77.00 ppm, CDCl_3_). The following abbreviations (or combinations thereof) were used to explain multiplicities: s = singlet, d = doublet, t = triplet, m = multiplet, br = broad, q = quadruplet. The melting point of solid compounds was determined by X-5 micro melting point tester.

### Synthesis of β-Halovinyl Ketones

PdCl_2_(PPh_3_)_2_ (2.8 mg, 0.02 equiv.), CuI (1.5 mg, 0.04 equiv.), Et_3_N (33.3 μL, 1.2 equiv.), alkynes (0.2 mmol), acid halides (0.26 mmol), and DCE (0.5 mL) were added sequentially under N_2_ to a reaction tube (10 mL), equipped with a magnetic stir bar. Then the resulting mixture was stirred at room temperature for 10 min. HOTf (26.5 μL, 1.5 equiv.) was added to the reaction, which was stirred at room temperature for 4 h. The mixture was filtered through silica gel and the filtrate was concentrated. The residue was purified by flash column chromatography to afford the desired product.

### Gram-Scale Synthesis of β-Chlorovinyl Ketone 3l

To a round bottom flask (100 mL), equipped with a magnetic stir bar, was added PdCl_2_(PPh_3_)_2_ (140.4 mg, 0.02 equiv.), CuI (76.2 mg, 0.04 equiv.), Et_3_N (1.7 mL, 1.2 equiv.), 3,3-dimethyl-1-butyne (1.2 mL, 10 mmol), benzoyl chloride (1.5 mL, 13 mmol), and 25 mL of DCE sequentially under N_2_ and then the resulting mixture was stirred at room temperature for 10 min. HOTf (1.3 mL, 1.5 equiv.) was added to the reaction, stirred at room temperature for 4 h. The mixture was filtered through silica gel and the filtrate was concentrated. EtOAc (30 mL) was added and the reaction mixture was washed with NaHCO_3_ (10 mL), water (2 × 10 mL), brine (10 mL) to remove excess acid. The organic layer was dried over anhydrous Na_2_SO_4_, and concentrated under reduced pressure. The residue was purified by flash column chromatography (silica gel, petroleum ether/ethyl acetate = 20/1) to afford the desired product **3l** as a pale yellow oil (1.66 g, 74%).

### Characterization Data

*(Z)-3-Chloro-1,3-diphenylprop-2-en-1-one (****3a****):* Prepared according to the general procedure. The crude reaction mixture was purified by flash column chromatography using PE/EA = 100/1 as the eluent to give 41.8 mg (87% yield) of **3a** (Z/E = 91/9) as a pale yellow oil; ^1^H NMR (400 MHz, CDCl_3_) δ 8.01 (d, *J* = 8.0 Hz, 2H), 7.77–7.75 (m, 2H), 7.61–7.58 (m, 1H), 7.53–7.42 (m, 5H), 7.36 (s, 1H); ^13^C NMR (100 MHz, CDCl3) δ 189.9, 143.3, 137.8, 137.3, 133.3, 130.5, 128.7, 128.7, 127.2, 121.5.

*(Z)-3-Chloro-3-(4-chlorophenyl)-1-phenylprop-2-en-1-one (****3b****):* Prepared according to the general procedure. The crude reaction mixture was purified by flash column chromatography using PE/EA = 100/1 as the eluent to give 45.7 mg (85% yield) of **3b** (Z/E = 85/15) as a pale yellow solid; m.p. 54–57°C; ^1^H NMR (500 MHz, CDCl_3_) δ 7.98 (d, *J* = 8.0 Hz, 2H), 7.69 (d, *J* = 8.0 Hz, 2H), 7.61–7.59 (m, 1H), 7.51–7.48 (m, 2H), 7.41 (d, *J* = 8.0 Hz, 2H), 7.33 (s, 1H); ^13^C NMR (125 MHz, CDCl3) δ 189.7, 141.8, 137.5, 136.7, 135.7, 133.4, 128.9, 128.7, 128.6, 128.4, 121.8.

*(Z)-3-Chloro*−*3-(3-chlorophenyl)-1-phenylprop-2-en-1-one (****3c****):* Prepared according to the general procedure. The crude reaction mixture was purified by flash column chromatography using PE/EA = 100/1 as the eluent to give 44.2 mg (86% yield) of **3c** (Z/E = 91/9) as a pale yellow solid; m.p. 84-86 °C; ^1^H NMR (500 MHz, CDCl_3_) δ 8.00 (d, *J* = 7.5 Hz, 2H), 7.74 (s, 1H), 7.64 (d, *J* = 8.0 Hz, 1H), 7.60 (d, *J* = 7.0 Hz, 1H), 7.52–7.49 (m, 2H), 7.44 (d, *J* = 8.0 Hz, 1H), 7.41–7.37 (m, 1H), 7.33 (s, 1H); ^13^C NMR (125 MHz, CDCl3) δ 189.7, 141.2, 139.0, 137.4, 134.8, 133.5, 130.5, 129.9, 128.8, 128.7, 127.3, 125.3, 122.6. HRMS (ESI): m/z calcd. for C_15_H_10_Cl_2_O [M + Na]^+^ 299.0001, found 299.0002.

*(Z)-3-Chloro-3-(4-fluorophenyl)-1-phenylprop-2-en-1-one (****3d***): Prepared according to the general procedure. The crude reaction mixture was purified by flash column chromatography using PE/EA = 100/1 as the eluent to give 39.5 mg (76% yield) of **3d** (Z/E = 89/11) as a pale yellow oil; ^1^H NMR (500 MHz, CDCl_3_) δ 8.00 (d, *J* = 5.5 Hz, 2H), 7.77–7.75 (m, 2H), 7.64–7.56 (m, 1H), 7.55–7.46 (m, 2H), 7.31 (s, 1H), 7.15–7.12 (m, 2H); ^13^C NMR (125 MHz, CDCl_3_) δ 189.7, 164.0 (d, *J* = 250.0 Hz), 142.1, 137.6, 133.5 (d, *J* = 3.8 Hz), 133.4, 129.2 (d, *J* = 8.8 Hz), 128.7, 128.6, 121.4, 115.7 (d, *J* = 21.3 Hz).

*(Z)-3-(4-Bromophenyl)-3-chloro-1-phenylprop-2-en-1-one (****3e****):* Prepared according to the general procedure. The crude reaction mixture was purified by flash column chromatography using PE/EA = 100/1 as the eluent to give 53.9 mg (84% yield) of **3e** (Z/E = 87/13) as a pale yellow oil; ^1^H NMR (500 MHz, CDCl_3_) δ 7.99 (d, *J* = 8.0 Hz, 2H), 7.66-7.54 (m, 5H), 7.51–7.48 (m, 2H), 7.33 (s, 1H); ^13^C NMR (125 MHz, CDCl_3_) δ 189.7, 141.9, 137.5, 136.2, 133.4, 131.9, 128.7, 128.6, 128.6, 125.0, 121.9.

*(Z)-3-Chloro-1-phenyl-3-(p-tolyl)prop-2-en-1-one (****3f****):* Prepared according to the general procedure. The crude reaction mixture was purified by flash column chromatography using PE/EA = 100/1 as the eluent to give 40.9 mg (81% yield) of **3f** (Z/E = 93/7) as a pale yellow oil; ^1^H NMR (500 MHz, CDCl3) δ 7.99 (d, *J* = 8.0 Hz, 2H), 7.66 (d, *J* = 8.0 Hz, 2H), 7.59–7.56 (m, 1H), 7.50–7.47 (m, 2H), 7.35 (s, 1H), 7.24 (d, *J* = 8.0 Hz, 2H), 2.40 (s, 3H); ^13^C NMR (125 MHz, CDCl_3_) δ 189.8, 143.6, 141.0, 137.9, 134.5, 133.2, 129.3, 128.6, 128.6, 127.1, 120.4, 21.3.

*(Z)-3-Chloro-3-(4-methoxyphenyl)-1-phenylprop-2-en-1-one (****3g****):* Prepared according to the general procedure. The crude reaction mixture was purified by flash column chromatography using PE/EA = 80/1 as the eluent to give 32 mg (57% yield) of **3g** (Z/E = 89/11) as a pale yellow oil; ^1^H NMR (400 MHz, CDCl_3_) δ 7.99 (d, *J* = 7.2 Hz, 2H), 7.73 (d, *J* = 8.8 Hz, 2H), 7.60–7.56 (m, 1H), 7.51–7.47 (m, 2H), 7.32 (s, 1H), 6.95 (d, *J* = 9.2 Hz, 2H), 3.87 (s, 3H); ^13^C NMR (100 MHz, CDCl_3_) δ 189.6, 161.6, 143.8, 138.2, 133.1, 129.7, 128.8, 128.6, 128.5, 119.2, 114.0, 55.5.

*(Z)-3-Chloro-4-hydroxy-4-methyl-1-phenylpent-2-en-1-one (****3h****):* Prepared according to the general procedure. The crude reaction mixture was purified by flash column chromatography using PE/EA = 10/1 as the eluent to give 24.9 mg (39% yield) of **3h** (Z/E > 99/1) as a pale yellow solid; ^1^H NMR (400 MHz, CDCl_3_) δ 7.83 (d, *J* = 7.2 Hz, 2H), 7.58–7.54 (m, 1H), 7.51–7.47 (m, 2H), 5.97 (s, 1H), 1.49 (s, 6H); ^13^C NMR (100 MHz, CDCl_3_) δ 207.0, 183.4, 132.6, 129.2, 128.8, 127.1, 98.6, 89.0, 23.1. HRMS (ESI): m/z calcd. for C_12_H_13_ClO_2_ [M + Na]^+^ 225.0677, found 225.0657.

*(Z)-3-Chloro-1-phenyl-3-(thiophen-3-yl)prop-2-en-1-one (****3i****):* Prepared according to the general procedure. The crude reaction mixture was purified by flash column chromatography using PE/EA = 100/1 as the eluent to give 37.4 mg (75% yield) of **3i** (Z/E = 92/8) as pale yellow oil; ^1^H NMR (400 MHz, CDCl_3_) δ 7.98 (d, *J* = 7.2 Hz, 2H), 7.85–7.83 (m, 1H), 7.60–7.57 (m, 1H), 7.47-7.51 (m, 2H), 7.43–7.41 (m, 1H), 7.41–7.39 (m, 1H), 7.37 (s, 1H); ^13^C NMR (100 MHz, CDCl_3_) δ 189.6, 139.3, 138.0, 137.9, 133.2, 128.7, 128.5, 127.7, 127.1, 125.0, 119.3. HRMS (ESI): m/z calcd. for C_13_H_9_ClOS [M + Na]^+^ 270.9955, found 270.9939.

*(Z)-3-Chloro-3-(1-hydroxycyclohexyl)-1-phenylprop-2-en-1-one (****3j****):* Prepared according to the general procedure. The crude reaction mixture was purified by flash column chromatography using PE/EA = 10/1 as the eluent to give 26.0 mg (49% yield) of **3j** (Z/E > 99/1) as a pale yellow oil; ^1^H NMR (400 MHz, CDCl_3_) δ 7.85 (d, *J* = 7.2 Hz, 2H), 7.58–7.54 (m, 1H), 7.51–7.46 (m, 2H), 5.98 (s, 1H), 1.97–1.52 (m, 10H); ^13^C NMR (100 MHz, CDCl_3_) δ 206.9, 183.4, 132.5, 129.3, 128.8, 127.1, 99.2, 90.8, 31.9, 24.5, 21.9. HRMS (ESI): m/z calcd. for C_15_H_17_ClO_2_ [M + H]^+^ 265.0990, found 265.1009.

*(Z)-3-Chloro-1-phenylhept-2-en-1-one (****3k****):* Prepared according to the general procedure. The crude reaction mixture was purified by flash column chromatography using PE/EA = 100/1 as the eluent to give 28.4 mg (63% yield) of **3k** (Z/E = 41/59) as a pale yellow oil; ^1^H NMR (400 MHz, CDCl_3_) δ 7.93 (d, *J* = 7.0 Hz, 2H), 7.59–7.55 (m, 1H), 7.49–7.45 (m, 2H), 6.82 (s, 1H), 2.55 (t, *J* = 8.0 Hz, 2H), 1.72–1.65 (m, 2H), 1.47–1.37 (m, 2H), 0.97 (t, *J* = 8.0 Hz, 3H); ^13^C NMR (100 MHz, CDCl_3_) δ 188.6, 157.8, 138.1, 133.0, 128.7, 128.3, 123.3, 36.2, 29.9, 22.1, 13.8.

*(Z)-3-Chloro-4,4-dimethyl-1-phenylpent-2-en-1-one (****3l****):* Prepared according to the general procedure. The crude reaction mixture was purified by flash column chromatography using PE/EA = 20/1 as the eluent to give 33.0 mg (75% yield) of **3l** (Z/E > 99/1) as a pale yellow oil; ^1^H NMR (400 MHz, CDCl_3_) δ 7.92 (d, *J* = 7.2 Hz, 2H), 7.59-7.55 (m, 1H), 7.49–7.45 (m, 2H), 6.72 (s, 1H), 1.28 (s, 9H); ^13^C NMR (100.0 MHz, CDCl_3_) δ 191.6, 154.6, 137.3, 133.2, 128.8, 128.6, 119.5, 39.9, 28.7.

*(Z)-3-Chloro-1-phenyl-3-(trimethylsilyl)prop-2-en-1-one (****3m****):* Prepared according to the general procedure. The crude reaction mixture was purified by flash column chromatography using PE/EA = 20/1 as the eluent to give 30.4 mg (63% yield) of **3m** (Z/E = 89/11) as a colorless oil; ^1^H NMR (400 MHz, CDCl_3_) δ 7.93 (d, *J* = 7.2 Hz, 2H), 7.61–7.57 (m, 1H), 7.50–7.47 (m, 2H), 7.04 (s, 1H), 0.31 (s, 9H); ^13^C NMR (100 MHz, CDCl_3_) δ 191.4, 148.6, 136.7, 133.5, 133.4, 128.9, 128.7,−2.5. HRMS (ESI): m/z calcd. for C_12_H_15_ClOSi [M + Na]^+^ 261.0473, found 261.0477.

*(Z)-3-Chloro-3-phenyl-1-(p-tolyl)prop-2-en-1-one (****4a****):* Prepared according to the general procedure. The crude reaction mixture was purified by flash column chromatography using PE/EA = 100/1 as the eluent to give 46.6 mg (87% yield) of **4a** (Z/E = 93/7) as a pale yellow solid; m.p.: 71–73°C; ^1^H NMR (400 MHz, CDCl_3_) δ 7.91 (d, *J* = 8.0 Hz, 2H), 7.81–7.73 (m, 2H), 7.45–7.43 (m, 3H), 7.33 (s, 1H), 7.29 (d, *J* = 8.0 Hz, 2H), 2.43 (s, 3H); ^13^C NMR (100 MHz, CDCl_3_) δ 189.6, 144.3, 142.6, 137.3, 135.1, 130.4, 129.4, 128.8, 128.6, 127.1, 121.8, 21.7.

*(Z)-3-Chloro-1-(4-methoxyphenyl)-3-phenylprop-2-en-1-one1 (****4b****):* Prepared according to the general procedure. The crude reaction mixture was purified by flash column chromatography using PE/EA = 100/1 as the eluent to give 40.5 mg (75% yield) of **4b** (Z/E = 91/9) as a pale yellow oil; ^1^H NMR (400 MHz, CDCl_3_) δ 8.00 (d, *J* = 8.8 Hz, 2H), 7.76–7.74 (m, 2H), 7.45–7.43 (m, 3H), 7.29 (s, 1H), 6.96 (d, *J* = 8.8 Hz, 2H), 3.88 (s, 3H); ^13^C NMR (100 MHz, CDCl_3_) δ 188.7, 163.8, 141.9, 137.3, 131.1, 130.5, 130.3, 128.6, 127.0, 122.0, 113.9, 55.5.

*(Z)-3-Chloro-4,4-dimethyl-1-(p-tolyl)pent-2-en-1-one (****4c****):* Prepared according to the general procedure. The crude reaction mixture was purified by flash column chromatography using PE/EA = 50/1 as the eluent to give 40.7 mg (92% yield) of **4c** (Z/E > 99/1) as a pale yellow oil; ^1^H NMR (400 MHz, CDCl_3_) δ 7.83 (d, *J* = 8.4 Hz, 2H), 7.27–7.25 (m, 2H), 6.68 (s, 1H), 2.42 (s, 3H), 1.32 (s, 6H); ^13^C NMR (100 MHz, CDCl_3_) δ 191.4, 153.9, 144.2, 134.8, 129.3, 129.0, 119.7, 39.8, 28.7, 21.7. HRMS (ESI): m/z calcd. for C_14_H_17_ClO [M+H]^+^ 237.1041, Found 237.1048.

*(Z)-3-chloro-4,4-dimethyl-1-(m-tolyl)pent-2-en-1-one (****4d****):* Prepared according to the general procedure. The crude reaction mixture was purified by flash column chromatography using PE/EA = 20/1 as the eluent to give 38.8 mg (81% yield) of **4d** (Z/E > 99/1) as a pale yellow oil; ^1^H NMR (400 MHz, CDCl_3_) δ 7.74 (s, 1H), 7.70 (d, *J* = 7.2 Hz, 1H), 7.44–7.29 (m, 2H), 6.71 (s, 1H), 2.41 (s, 3H), 1.32 (s, 9H); ^13^C NMR (100 MHz, CDCl_3_) δ 191.8, 154.4, 138.4, 137.3, 134.0, 129.1, 128.4, 126.1, 119.6, 39.8, 28.6, 21.3. HRMS (ESI): m/z calcd. for C_14_H_17_ClO [M+H]^+^ 237.1041, found 237.1053.

*(Z)-3-Chloro-1-(4-methoxyphenyl)-4,4-dimethylpent-2-en-1-one (****4e****):* Prepared according to the general procedure. The crude reaction mixture was purified by flash column chromatography using PE/EA = 30/1 as the eluent to give 31.1 mg (63% yield) of **4e** (Z/E > 99/1) as a pale yellow oil; ^1^H NMR (400 MHz, CDCl_3_) δ 7.91 (d, *J* = 8.8 Hz, 2H), 6.94 (d, *J* = 8.8 Hz, 2H), 6.63 (s, 1H), 3.87 (s, 2H), 1.30 (s, 9H); ^13^C NMR (100 MHz, CDCl_3_) δ 190.6, 163.7, 153.2, 131.3, 130.2, 119.9, 113.8, 55.5, 39.7, 28.7. HRMS (ESI): m/z calcd. for C_14_H_17_ClO_2_ [M + Na]^+^ 275.0809, found 275.0805.

*(Z)-3-Chloro-4,4-dimethyl-1-(o-tolyl)pent-2-en-1-one (****4f****):* Prepared according to the general procedure. The crude reaction mixture was purified by flash column chromatography using PE/EA = 30/1 as the eluent to give 41 mg (89% yield) of **4f** (Z/E > 99/1) as a pale yellow oil; ^1^H NMR (400 MHz, CDCl_3_) δ 7.57 (d, *J* = 7.2 Hz, 1H), 7.40–7.36 (m, 1H), 7.27–7.24 (m, 2H), 6.61 (s, 1H), 2.53 (s, 3H), 1.29 (s, 9H); ^13^C NMR (100 MHz, CDCl_3_) δ 194.6, 154.8, 138.4, 138.1, 131.7, 131.4, 129.5, 125.6, 121.8, 39.9, 28.6, 20.9. HRMS (ESI): m/z calcd. for C_14_H_17_ClO [M + Na]^+^ 259.0860, found 259.0858.

*(Z)-3-Chloro-1-(4-fluorophenyl)-4,4-dimethylpent-2-en-1-one (****4g****):* Prepared according to the general procedure. The crude reaction mixture was purified by flash column chromatography using PE/EA = 20/1 as the eluent to give 42.4 mg (88% yield) of **4g** (Z/E > 99/1) as a pale yellow solid; m.p. 54–55°C; ^1^H NMR (400 MHz, CDCl_3_) δ 7.97–7.93 (m, 2H), 7.17–7.12 (m, 2H), 6.65 (s, 1H), 1.31 (s, 9H); ^13^C NMR (100 MHz, CDCl_3_) δ 190.2, 165.9 (d, *J* = 254 Hz), 154.7, 133.7 (d, *J* = 2.8 Hz), 131.5 (d, *J* = 9.4 Hz), 119.3, 115.8 (d, *J* = 22.0 Hz), 39.9, 28.6. HRMS (ESI): m/z calcd. for C_13_H_14_ClFO [M + Na]^+^ 263.0609, found 263.0613.

*(Z)-3-Chloro-1-(4-chlorophenyl)-4,4-dimethylpent-2-en-1-one (****4h****):* Prepared according to the general procedure. The crude reaction mixture was purified by flash column chromatography using PE/EA = 20/1 as the eluent to give 40.9 mg (80% yield) of **4h** (Z/E > 99/1) as a pale yellow solid; m.p. 64–66°C; ^1^H NMR (400 MHz, CDCl_3_) δ 7.90 (d, *J* = 8.4 Hz, 2H), 7.48 (d, *J* = 8.4 Hz, 2H), 6.70 (s, 1H), 1.36 (s, 9H); ^13^C NMR (100 MHz, CDCl_3_) δ 190.4, 155.2, 139.7, 135.6, 130.2, 128.9, 119.0, 39.9, 28.6. HRMS (ESI): m/z calcd. for C_13_H_14_Cl_2_O [M + Na]^+^ 279.0314, found 279.0327.

*(Z)-1-(4-Bromophenyl)-3-chloro-4,4-dimethylpent-2-en-1-one (****4i****):* Prepared according to the general procedure. The crude reaction mixture was purified by flash column chromatography using PE/EA = 20/1 as the eluent to give 41 mg (68% yield) of **4i** (Z/E > 99/1) as a pale yellow solid; m.p. 68–69°C; ^1^H NMR (400 MHz, CDCl_3_) δ 7.77 (d, *J* = 8.4 Hz, 2H), 7.60 (d, *J* = 8.4 Hz, 2H), 6.65 (s, 1H), 1.31 (s, 9H); ^13^C NMR (100 MHz, CDCl_3_) δ 190.6, 155.3, 136.1, 131.9, 130.3, 128.4, 119.0, 40.0, 28.6. HRMS (ESI): m/z calcd. for C_13_H_14_BrClO [M + Na]^+^ 322.9809, found 322.9795.

*(Z)-3-Chloro-1-(3-chlorophenyl)-4,4-dimethylpent-2-en-1-one (****4j****):* Prepared according to the general procedure. The crude reaction mixture was purified by flash column chromatography using PE/EA = 20/1 as the eluent to give 42.4 mg (82% yield) of **4j** (Z/E > 99/1) as a pale yellow solid; m.p. 55–56°C; ^1^H NMR (400 MHz, CDCl_3_) δ 7.88–7.87 (m, 1H), 7.77 (d, *J* = 8.0 Hz, 2H), 7.55–7.53 (m, 2H), 7.43–7.39 (m, 1H), 6.68 (s, 1H), 1.31 (s, 9H); ^13^C NMR (100 MHz, CDCl_3_) δ 190.2, 167.1, 164.6, 154.7, 131.5, 131.4, 119.3, 115.9, 115.6, 39.9, 28.6. HRMS (ESI): m/z calcd. for C_13_H_14_Cl_2_O [M + Na]^+^ 279.0314, found 279.0323.

*(Z)-1-(2-Bromophenyl)-3-chloro-4,4-dimethylpent-2-en-1-one (****4k****):* Prepared according to the general procedure. The crude reaction mixture was purified by flash column chromatography using PE/EA = 20/1 as the eluent to give 43.5 mg (72% yield) of **4k** (Z/E > 99/1) as a pale yellow solid; m.p. 64–66°C; ^1^H NMR (400 MHz, CDCl_3_) δ 7.59–7.61 (m, 1H), 7.49–7.46 (m, 1H), 7.40-7.36 (m, 1H), 7.33–7.29 (m, 1H), 6.68 (s, 1H), 1.27 (s, 9H); ^13^C NMR (100 MHz, CDCl_3_) δ 192.5, 157.4, 141.6, 133.5, 131.8, 129.9, 127.5, 121.2, 119.5, 40.3, 28.5. HRMS (ESI): m/z calcd. for C_13_H_14_BrClO [M + Na]^+^ 322.9809, found 322.9804.

*(Z)-4-(3-chloro-3-phenylacryloyl)benzonitrile (****4l****):* Prepared according to the general procedure. The crude reaction mixture was purified by prep-TLC using PE/EA = 10/1 as the eluent to give 14.3 mg (27% yield) of **4l** (Z/E > 99/1) as a yellow solid; m. p. 108.8–110.6°C; ^1^H NMR (500 MHz, CDCl_3_) δ 8.08 (d, *J* = 8.4 Hz, 2H), 7.84–7.73 (m, 4H), 7.54–7.44 (m, 3H), 7.33 (s, 1H); ^13^C NMR (126 MHz, CDCl_3_) δ 188.3, 145.8, 141.0, 136.9, 132.6, 131.3, 129.0, 128.8, 127.3, 120.2, 117.9, 116.4.

*(Z)-3-chloro-3-phenyl-1-(4-(trifluoromethyl)phenyl)prop-2-en-1-one (****4m****):* Prepared according to the general procedure. The crude reaction mixture was purified by prep-TLC using PE/EA = 10/1 as the eluent to give 37.4 mg (60% yield) of **4m** (Z/E > 99/1) as a pale yellow solid; m.p. 80.7–81.7°C; ^1^H NMR (500 MHz, CDCl_3_) δ 8.09 (d, *J* = 8.0 Hz, 2H), 7.83–7.68 (m, 4H), 7.53–7.40 (m, 3H), 7.35 (s, 1H); ^13^C NMR (126 MHz, CDCl_3_) δ 188.7, 145.1, 140.6,137.1, 134.5 (q, *J* = 32.8 Hz), 131.0, 128.9, 128.8, 127.3, 125.8 (q, *J* = 3.8 Hz), 123.6 (q, *J* = 273.4 Hz), 120.6.

*(Z)-3-Chloro-1-cyclohexyl-4,4-dimethylpent-2-en-1-one (****4n****):* Prepared according to the general procedure. The crude reaction mixture was purified by flash column chromatography using PE/EA = 20/1 as the eluent to give 35.9 mg (82% yield) of **4n** (Z/E > 99/1) as a colorless oil; ^1^H NMR (400 MHz, CDCl_3_) δ 6.31 (s, 1H), 2.56–2.50 (m, 1H), 1.87–1.75 (m, 6H), 1.43-1.25 (m, 4H), 1.23 (s, 9H); ^13^C NMR (100 MHz, CDCl_3_) δ 203.1, 154.6, 120.3, 51.3, 40.0, 28.7, 28.3, 25.8, 25.6. HRMS (ESI): m/z calcd. for C_13_H_21_ClO [M + Na]^+^ 251.1173, found 251.1174.

*(Z)-3-Chloro-4,4-dimethyl-1-(thiophen-2-yl)pent-2-en-1-one (****4o****):* Prepared according to the general procedure. The crude reaction mixture was purified by flash column chromatography using PE/EA = 20/1 as the eluent to give 34.3 mg (77% yield) of **4o** (Z/E > 99/1) as a pale yellow oil; ^1^H NMR (400 MHz, CDCl_3_) δ 7.72–7.58 (m, 2H), 7.15–7.13 (m, 1H), 6.76 (s, 1H), 1.30 (s, 9H); ^13^C NMR (100 MHz, CDCl_3_) δ 182.8, 156.0, 145.0, 134.1, 132.4, 128.1, 118.4, 40.1, 28.6. HRMS (ESI): m/z calcd. for C_11_H_13_ClOS [M + Na]^+^ 251.0268, found 251.0274.

*(Z)-3-Bromo-4,4-dimethyl-1-phenylpent-2-en-1-one (****6****):* Prepared according to the general procedure. The crude reaction mixture was purified by flash column chromatography using PE/EA = 20/1 as the eluent to give 39.2 mg (70% yield) of **6** (Z/E > 99/1) as a pale yellow solid; m.p. 82–84°C; ^1^H NMR (400 MHz, CDCl_3_) δ 7.92 (d, *J* = 7.2 Hz, 2H), 7.60–7.56 (m, 1H), 7.50–7.46 (m, 2H), 6.91 (s, 1H), 1.31 (s, 9H); ^13^C NMR (100 MHz, CDCl_3_) δ 192.6, 147.5, 136.6, 133.4, 129.0, 128.6, 123.2, 40.5, 29.3.

## Data Availability Statement

The original contributions presented in the study are included in the article/Supplementary Material, further inquiries can be directed to the corresponding author/s.

## Author Contributions

YX designed the research. F-JC and ZH carried out the experiments. All authors contributed to the manuscript.

## Conflict of Interest

The authors declare that the research was conducted in the absence of any commercial or financial relationships that could be construed as a potential conflict of interest.
