# Supplementary material for: One-Pot Synthesis of (Z)-β-Halovinyl Ketones via the Cascade of Sonogashira Coupling and Hydrohalogenation
Source: Front Chem. 2021 Apr 22;8:621545. doi: 10.3389/fchem.2020.621545 (PMC8100659; doi:10.3389/fchem.2020.621545)
Supplement: Supplementary file 1 [file Data_Sheet_1.docx]

**Supporting Information**

One-Pot Synthesis of (*Z*)-β-Halovinyl Ketones via the Cascade of Sonogashira Coupling and Hydrohalogenation

Fa-Jie Chen,^†a, b^ Zhenguo Hua,^†a^ Jianhui Chen, ^a^ Jiajia Chen, ^a^ Daesung Lee,* ^b^ and Yuanzhi Xia*^a^

*^a^*College of Chemistry and Materials Engineering, Wenzhou University, Wenzhou 325035, P. R. China. Email: xyz@wzu.edu.cn

*^b^*Department of Chemistry, University of Illinois at Chicago, 845 West Taylor Street, Chicago, IL 60607 (USA). Email：dsunglee@uic.edu

^†^These authors contributed equally.

##

Table of Contents Pages

[I. General Information S2](#_Toc45546855)

[II. General Procedure for the Synthesis of β-Halovinyl Ketones S3](#_Toc45546856)

[III. Gram-scale Synthesis of β-Chlorovinyl Ketone](#_Toc45546857) **[3l](#_Toc45546857)** [S3](#_Toc45546857)

[IV. Characterization Data S4](#_Toc45546858)

[V. References S18](#_Toc45546859)

[VI.](#_Toc45546860) ^[1](#_Toc45546860)^[H and](#_Toc45546860) ^[13](#_Toc45546860)^[C NMR Spectra S19](#_Toc45546860)

## I. General Information

Unless otherwise noted, all chemicals were purchased from J&K, Energy-Chemical or Tansoole, and used as received. All reactions were carried out using oven-dried glassware and magnetic stirring under N_2_. The product was monitored and tracked by TLC (iodine, potassium permanganate, and other color reagents will be used if necessary). The product was extracted and filtered with 100-200 mesh silica gel and separated and purified with chromatography silica gel column or chromatography silica gel plate (specification of silica gel is 300-400 mesh silica gel). ^1^H NMR spectrum and ^13^C NMR spectrum were determined by Bruker-400 instrument or Bruker-500 instrument with TMS as internal standard and deuterium chloroform as solvent. ^1^H NMR chemical shifts were referenced to tetramethylsilane signal (0 ppm), ^13^C NMR chemical shifts were referenced to the solvent resonance (77.00 ppm, CDCl_3_). The following abbreviations (or combinations thereof) were used to explain multiplicities: s = singlet, d = doublet, t = triplet, m = multiplet, br = broad, q = quadruplet. The melting point of solid compounds was determined by X-5 micro melting point tester.

## II. General Procedure for the Synthesis of β-Halovinyl Ketones

PdCl_2_(PPh_3_)_2_ (2.8 mg, 0.02 equiv.), CuI (1.5 mg, 0.04 equiv.), Et_3_N (33.3 μL, 1.2 equiv.), alkynes (0.2 mmol), acid halides (0.26 mmol), and DCE (0.5 mL) were added sequentially under N_2_ to a reaction tube (10 mL), equipped with a magnetic stir bar. Then the resulting mixture was stirred at room temperature for 10 min. HOTf (26.5 μL, 1.5 equiv.) was added to the reaction, which was stirred at room temperature for 4 h. The mixture was filtered through silica gel and the filtrate was concentrated. The residue was purified by flash column chromatography to afford the desired product.

## III. Gram-scale Synthesis of β-Chlorovinyl Ketone 3l

To a round bottom flask (100 mL), equipped with a magnetic stir bar, was added PdCl_2_(PPh_3_)_2_ (140.4 mg, 0.02 equiv.), CuI (76.2 mg, 0.04 equiv.), Et_3_N (1.7 mL, 1.2 equiv.), [3,3-dimethyl-1-butyne](javascript:showMsgDetail('ProductSynonyms.aspx?CBNumber=CB9228568&postData3=CN&SYMBOL_Type=A');) (1.2 mL, 10 mmol), [benzoyl chloride](javascript:showMsgDetail('ProductSynonyms.aspx?CBNumber=CB8854753&postData3=CN&SYMBOL_Type=A');) (1.5 mL, 13 mmol), and 25 mL of DCE sequentially under N_2_ and then the resulting mixture was stirred at room temperature for 10 min. HOTf (1.3 mL, 1.5 equiv.) was added to the reaction, stirred at room temperature for 4 h. The mixture was filtered through silica gel and the filtrate was concentrated. EtOAc (30 mL) was added and the reaction mixture was washed with NaHCO_3_ (10 mL), water (2 × 10 mL), brine (10 mL) to remove excess acid. The organic layer was dried over anhydrous Na_2_SO_4_, and concentrated under reduced pressure. The residue was purified by flash column chromatography (silica gel, PE/EtOAc = 20/1) to afford the desired product **3l** as pale yellow oil (1.66 g, 74%).

## IV. Characterization Data

**(*Z*)-3-Chloro-1,3-diphenylprop-2-en-1-one^1^ (3a)**

Prepared according to the general procedure using PdCl_2_(PPh_3_)_2_ (2.8 mg, 0.02 equiv.), CuI (1.5 mg, 0.04 equiv.), Et_3_N (33.3 μL, 1.2 equiv.), phenylacetylene (21.7 μL, 0.2 mmol) and benzoyl chloride (30.2 μL, 0.26 mmol), and DCE (0.5 mL) sequentially under N_2_ and then the resulting mixture was stirred at room temperature for 10 min. HOTf (26.5 μL, 1.5 equiv.) was added to the reaction, stirred at room temperature for 4 h. The crude reaction mixture was purified by flash column chromatography using PE/EA = 100/1 as the eluent to give 41.8 mg (87% yield) of **3a** (Z/E = 91/9) as a pale yellow oil; ^1^H NMR (400 MHz, CDCl_3_) δ 8.01 (d, *J* = 8.0 Hz, 2H), 7.77-7.75 (m, 2H), 7.61-7.58 (m, 1H), 7.53-7.42 (m, 5H), 7.36 (s, 1H); ^13^C NMR (100 MHz, CDCl_3_) δ 189.9, 143.3, 137.8, 137.3, 133.3, 130.5, 128.7, 128.7, 127.2, 121.5.

**(*Z*)-3-Chloro-3-(4-chlorophenyl)-1-phenylprop-2-en-1-one^1^ (3b)**

Prepared according to the general procedure using PdCl_2_(PPh_3_)_2_ (2.8 mg, 0.02 equiv.), CuI (1.5 mg, 0.04 equiv.), Et_3_N (33.3 μL, 1.2 equiv.), 1-chloro-4-ethynylbenzene (27.3 mg, 0.2 mmol) and benzoyl chloride (30.2 μL, 0.26 mmol) , and DCE (0.5 mL) sequentially under N_2_ and then the resulting mixture was stirred at room temperature for 10 min. HOTf (26.5 μL, 1.5 equiv.) was added to the reaction, stirred at room temperature for 4 h. The crude reaction mixture was purified by flash column chromatography using PE/EA = 100/1 as the eluent to give 45.7 mg (85% yield) of **3b** (Z/E = 85/15) as a pale yellow solid; mp 54-57 ^o^C; ^1^H NMR (500 MHz, CDCl_3_) δ 7.98 (d, *J* = 8.0 Hz, 2H), 7.69 (d, *J* = 8.0 Hz, 2H), 7.61-7.59 (m, 1H), 7.51-7.48 (m, 2H), 7.41 (d, *J* = 8.0 Hz, 2H), 7.33 (s, 1H); ^13^C NMR (125 MHz, CDCl_3_) δ 189.7, 141.8, 137.5, 136.7, 135.7, 133.4, 128.9, 128.7, 128.6, 128.4, 121.8.

**(*Z*)-3-Chloro -3-(3-chlorophenyl)-1-phenylprop-2-en-1-one (3c)**

Prepared according to the general procedure using PdCl_2_(PPh_3_)_2_ (2.8 mg, 0.02 equiv.), CuI (1.5 mg, 0.04 equiv.), Et_3_N (33.3 μL, 1.2 equiv.), 1-chloro-3-ethynylbenzene (27.3 mg, 0.2 mmol) and benzoyl chloride (30.2 μL, 0.26 mmol) , and DCE (0.5 mL) sequentially under N_2_ and then the resulting mixture was stirred at room temperature for 10 min. HOTf (26.5 μL, 1.5 equiv.) was added to the reaction, stirred at room temperature for 4 h. The crude reaction mixture was purified by flash column chromatography using PE/EA = 100/1 as the eluent to give 44.2 mg (86% yield) of **3c** (Z/E = 91/9) as a pale yellow solid; mp 84-86 ^o^C; ^1^H NMR (500 MHz, CDCl_3_) δ 8.00 (d, *J* = 7.5 Hz, 2H), 7.74 (s, 1H), 7.64 (d, *J* = 8.0 Hz, 1H), 7.60 (d, *J* = 7.0 Hz, 1H), 7.52-7.49 (m, 2H), 7.44 (d, *J* = 8.0 Hz, 1H), 7.41-7.37 (m, 1H), 7.33 (s, 1H); ^13^C NMR (125 MHz, CDCl_3_) δ 189.7, 141.2, 139.0, 137.4, 134.8, 133.5, 130.5, 129.9, 128.8, 128.7, 127.3, 125.3, 122.6. HRMS (ESI): m/z calcd. for C_15_H_10_Cl_2_O [M + Na]^+^ 299.0001, found 299.0002.

**(*Z*)-3-Chloro-3-(4-fluorophenyl)-1-phenylprop-2-en-1-one^2^ (3d)**

Prepared according to the general procedure using PdCl_2_(PPh_3_)_2_ (2.8 mg, 0.02 equiv.), CuI (1.5 mg, 0.04 equiv.), Et_3_N (33.3 μL, 1.2 equiv.), 1-ethynyl-4-fluorobenzene (24.0 mg, 0.2 mmol) and benzoyl chloride (30.2 μL, 0.26 mmol) , and DCE (0.5 mL) sequentially under N_2_ and then the resulting mixture was stirred at room temperature for 10 min. HOTf (26.5 μL, 1.5 equiv.) was added to the reaction, stirred at room temperature for 4 h. The crude reaction mixture was purified by flash column chromatography using PE/EA = 100/1 as the eluent to give 39.5 mg (76% yield) of **3d** (Z/E = 89/11) as a pale yellow oil; ^1^H NMR (500 MHz, CDCl_3_) δ 8.00 (d, *J* = 5.5 Hz, 2H), 7.77-7.75 (m, 2H), 7.64-7.56 (m, 1H), 7.55-7.46 (m, 2H), 7.31 (s, 1H), 7.15-7.12 (m, 2H); ^13^C NMR (125 MHz, CDCl_3_) δ 189.7, 164.0 (d, *J* = 250.0 Hz), 142.1, 137.6, 133.5 (d, *J* = 3.8 Hz), 133.4, 129.2 (d, *J* = 8.8 Hz), 128.7, 128.6, 121.4, 115.7 (d, *J* = 21.3 Hz).

**(*Z*)-3-(4-Bromophenyl)-3-chloro-1-phenylprop-2-en-1-one^2^ (3e)**

Prepared according to the general procedure using PdCl_2_(PPh_3_)_2_ (2.8 mg, 0.02 equiv.), CuI (1.5 mg, 0.04 equiv.), Et_3_N (33.3 μL, 1.2 equiv.), 1-bromo-4-ethynylbenzene (36.2 mg, 0.2 mmol) and benzoyl chloride (30.2 μL, 0.26 mmol), and DCE (0.5 mL) sequentially under N_2_ and then the resulting mixture was stirred at room temperature for 10 min. HOTf (26.5 μL, 1.5 equiv.) was added to the reaction, stirred at room temperature for 4 h. The crude reaction mixture was purified by flash column chromatography using PE/EA = 100/1 as the eluent to give 53.9 mg (84% yield) of **3e** (Z/E = 87/13) as a pale yellow oil; ^1^H NMR (500 MHz, CDCl_3_) δ 7.99 (d, *J* = 8.0 Hz, 2H), 7.66-7.54 (m, 5H), 7.51-7.48 (m, 2H), 7.33 (s, 1H); ^13^C NMR (125 MHz, CDCl_3_) δ 189.7, 141.9, 137.5, 136.2, 133.4, 131.9, 128.7, 128.6, 128.6, 125.0, 121.9.

**(*Z*)-3-Chloro-1-phenyl-3-(p-tolyl)prop-2-en-1-one^1^ (3f)**

Prepared according to the general procedure using PdCl_2_(PPh_3_)_2_ (2.8 mg, 0.02 equiv.), CuI (1.5 mg, 0.04 equiv.), Et_3_N (33.3 μL, 1.2 equiv.), 1-ethynyl-4-methylbenzene (23.2 mg, 0.2 mmol) and benzoyl chloride (30.2 μL, 0.26 mmol) , and DCE (0.5 mL) sequentially under N_2_ and then the resulting mixture was stirred at room temperature for 10 min. HOTf (26.5 μL, 1.5 equiv.) was added to the reaction, stirred at room temperature for 4 h. The crude reaction mixture was purified by flash column chromatography using PE/EA = 100/1 as the eluent to give 40.9 mg (81% yield) of **3f** (Z/E = 93/7) as a pale yellow oil; ^1^H NMR (500 MHz, CDCl_3_) δ 7.99 (d, J = 8.0 Hz, 2H), 7.66 (d, J = 8.0 Hz, 2H), 7.59-7.56 (m, 1H), 7.50-7.47 (m, 2H), 7.35 (s, 1H), 7.24 (d, J = 8.0 Hz, 2H), 2.40 (s, 3H); ^13^C NMR (125 MHz, CDCl_3_) δ 189.8, 143.6, 141.0, 137.9, 134.5, 133.2, 129.3, 128.6, 128.6, 127.1, 120.4, 21.3.

**(*Z*)-3-Chloro-3-(4-methoxyphenyl)-1-phenylprop-2-en-1-one^2^ (3g)**

Prepared according to the general procedure using PdCl_2_(PPh_3_)_2_ (2.8 mg, 0.02 equiv.), CuI (1.5 mg, 0.04 equiv.), Et_3_N (33.3 μL, 1.2 equiv.), 1-ethynyl-4-methoxybenzene (26.4 mg, 0.2 mmol) and benzoyl chloride (30.2 μL, 0.26 mmol) , and DCE (0.5 mL) sequentially under N_2_ and then the resulting mixture was stirred at room temperature for 10 min. HOTf (26.5 μL, 1.5 equiv.) was added to the reaction, stirred at room temperature for 4 h. The crude reaction mixture was purified by flash column chromatography using PE/EA = 80/1 as the eluent to give 32 mg (57% yield) of **3g** (Z/E = 89/11) as a pale yellow oil; ^1^H NMR (400 MHz, CDCl_3_) δ 7.99 (d, *J* = 7.2 Hz, 2H), 7.73 (d, *J* = 8.8 Hz, 2H), 7.60-7.56 (m, 1H), 7.51-7.47 (m, 2H), 7.32 (s, 1H), 6.95 (d, *J* = 9.2 Hz, 2H), 3.87 (s, 3H); ^13^C NMR (100 MHz, CDCl_3_) δ 189.6, 161.6, 143.8, 138.2, 133.1, 129.7, 128.8, 128.6, 128.5, 119.2, 114.0, 55.5.

**(*Z*)-3-Chloro-4-hydroxy-4-methyl-1-phenylpent-2-en-1-one (3h)**

Prepared according to the general procedure using PdCl_2_(PPh_3_)_2_ (2.8 mg, 0.02 equiv.), CuI (1.5 mg, 0.04 equiv.), Et_3_N (33.3 μL, 1.2 equiv.), 2-methylbut-3-yn-2-ol (19.4 μL, 0.2 mmol) and benzoyl chloride (30.2 μL, 0.26 mmol), and DCE (0.5 mL) sequentially under N_2_ and then the resulting mixture was stirred at room temperature for 10 min. HOTf (26.5 μL, 1.5 equiv.) was added to the reaction, stirred at room temperature for 4 h. The crude reaction mixture was purified by flash column chromatography using PE/EA = 10/1 as the eluent to give 24.9 mg (39% yield) of **3h** (Z/E > 99/1) as a pale yellow solid; ^1^H NMR (400 MHz, CDCl_3_) δ 7.83 (d, *J* = 7.2 Hz, 2H), 7.58-7.54 (m, 1H), 7.51-7.47 (m, 2H), 5.97 (s, 1H), 1.49 (s, 6H); ^13^C NMR (100 MHz, CDCl_3_) δ 207.0, 183.4, 132.6, 129.2, 128.8, 127.1, 98.6, 89.0, 23.1. HRMS (ESI): m/z calcd. for C_12_H_13_ClO_2_ [M + Na]^+^ 225.0677, found 225.0657.

**(*Z*)-3-Chloro-1-phenyl-3-(thiophen-3-yl)prop-2-en-1-one (3i)**

Prepared according to the general procedure using PdCl_2_(PPh_3_)_2_ (2.8 mg, 0.02 equiv.), CuI (1.5 mg, 0.04 equiv.), Et_3_N (33.3 μL, 1.2 equiv.), 3-ethynylthiophene (19.7 μL, 0.2 mmol) and benzoyl chloride (30.2 μL, 0.26 mmol), and DCE (0.5 mL) sequentially under N_2_ and then the resulting mixture was stirred at room temperature for 10 min. HOTf (26.5 μL, 1.5 equiv.) was added to the reaction, stirred at room temperature for 4 h. The crude reaction mixture was purified by flash column chromatography using PE/EA = 100/1 as the eluent to give 37.4 mg (75% yield) of **3i** (Z/E = 92/8) as pale yellow oil; ^1^H NMR (400 MHz, CDCl_3_) δ 7.98 (d, *J* = 7.2 Hz, 2H), 7.85-7.83 (m, 1H), 7.60-7.57 (m, 1H), 7.47-7.51 (m, 2H), 7.43-7.41 (m, 1H), 7.41-7.39 (m, 1H), 7.37 (s, 1H); ^13^C NMR (100 MHz, CDCl_3_) δ 189.6, 139.3, 138.0, 137.9, 133.2, 128.7, 128.5, 127.7, 127.1, 125.0, 119.3. HRMS (ESI): m/z calcd. for C_13_H_9_ClOS [M + Na]^+^ 270.9955, found 270.9939.

**(*Z*)-3-Chloro-3-(1-hydroxycyclohexyl)-1-phenylprop-2-en-1-one (3j)**

Prepared according to the general procedure using PdCl_2_(PPh_3_)_2_ (2.8 mg, 0.02 equiv.), CuI (1.5 mg, 0.04 equiv.), Et_3_N (33.3 μL, 1.2 equiv.), 1-ethynylcyclohexan-1-ol (25.6 μL, 0.2 mmol) and benzoyl chloride (30.2 μL, 0.26 mmol), and DCE (0.5 mL) sequentially under N_2_ and then the resulting mixture was stirred at room temperature for 10 min. HOTf (26.5 μL, 1.5 equiv.) was added to the reaction, stirred at room temperature for 4 h. The crude reaction mixture was purified by flash column chromatography using PE/EA = 10/1 as the eluent to give 26.0 mg (49% yield) of **3j** (Z/E > 99/1) as a pale yellow oil; ^1^H NMR (400 MHz, CDCl_3_) δ 7.85 (d, *J* = 7.2 Hz, 2H), 7.58-7.54 (m, 1H), 7.51-7.46 (m, 2H), 5.98 (s, 1H), 1.97-1.52 (m, 10H); ^13^C NMR (100 MHz, CDCl_3_) δ 206.9, 183.4, 132.5, 129.3, 128.8, 127.1, 99.2, 90.8, 31.9, 24.5, 21.9. HRMS (ESI): m/z calcd. for C_15_H_17_ClO_2_ [M + H]^+^ 265.0990, found 265.1009.

**(*Z*)-3-Chloro-1-phenylhept-2-en-1-one^1^ (3k)**

Prepared according to the general procedure using PdCl_2_(PPh_3_)_2_ (2.8 mg, 0.02 equiv.), CuI (1.5 mg, 0.04 equiv.), Et_3_N (33.3 μL, 1.2 equiv.), hex-1-yne (23 μL, 0.2 mmol) and benzoyl chloride (30.2 μL, 0.26 mmol), and DCE (0.5 mL) sequentially under N_2_ and then the resulting mixture was stirred at room temperature for 10 min. HOTf (26.5 μL, 1.5 equiv.) was added to the reaction, stirred at room temperature for 4 h. The crude reaction mixture was purified by flash column chromatography using PE/EA = 100/1 as the eluent to give 28.4 mg (63% yield) of **3k** (Z/E = 41/59) as a pale yellow oil; ^1^H NMR (400 MHz, CDCl_3_) δ 7.93 (d, *J* = 7.0 Hz, 2H), 7.59-7.55 (m, 1H), 7.49-7.45 (m, 2H), 6.82 (s, 1H), 2.55 (t, *J* = 8.0 Hz, 2H), 1.72-1.65 (m, 2H), 1.47-1.37 (m, 2H), 0.97 (t, *J* = 8.0 Hz, 3H); ^13^C NMR (100 MHz, CDCl_3_) δ 188.6, 157.8, 138.1, 133.0, 128.7, 128.3, 123.3, 36.2, 29.9, 22.1, 13.8.

**(*Z*)-3-Chloro-4,4-dimethyl-1-phenylpent-2-en-1-one^1^ (3l)**

Prepared according to the general procedure using PdCl_2_(PPh_3_)_2_ (2.8 mg, 0.02 equiv.), CuI (1.5 mg, 0.04 equiv.), Et_3_N (33.3 μL, 1.2 equiv.), 3,3-dimethylbut-1-yne (24.6 μL, 0.2 mmol) and benzoyl chloride (30.2 μL, 0.26 mmol), and DCE (0.5 mL) sequentially under N_2_ and then the resulting mixture was stirred at room temperature for 10 min. HOTf (26.5 μL, 1.5 equiv.) was added to the reaction, stirred at room temperature for 4 h. The crude reaction mixture was purified by flash column chromatography using PE/EA = 20/1 as the eluent to give 33.0 mg (75% yield) of **3l** (Z/E > 99/1) as a pale yellow oil; ^1^H NMR (400 MHz, CDCl_3_) δ 7.92 (d, *J* = 7.2 Hz, 2H), 7.59-7.55 (m, 1H), 7.49-7.45 (m, 2H), 6.72 (s, 1H), 1.28 (s, 9H); ^13^C NMR (100.0 MHz, CDCl_3_) δ 191.6, 154.6, 137.3, 133.2, 128.8, 128.6, 119.5, 39.9, 28.7.

**(*Z*)-3-Chloro-1-phenyl-3-(trimethylsilyl)prop-2-en-1-one (3m)**

Prepared according to the general procedure using PdCl_2_(PPh_3_)_2_ (2.8 mg, 0.02 equiv.), CuI (1.5 mg, 0.04 equiv.), Et_3_N (33.3 μL, 1.2 equiv.), ethynyltrimethylsilane (23 μL, 0.2 mmol) and benzoyl chloride (30.2 μL, 0.26 mmol), and DCE (0.5 mL) sequentially under N_2_ and then the resulting mixture was stirred at room temperature for 10 min. HOTf (26.5 μL, 1.5 equiv.) was added to the reaction, stirred at room temperature for 4 h. The crude reaction mixture was purified by flash column chromatography using PE/EA = 20/1 as the eluent to give 30.4 mg (63% yield) of **3m** (Z/E = 89/11) as a colorless oil; ^1^H NMR (400 MHz, CDCl_3_) δ 7.93 (d, *J* = 7.2 Hz, 2H), 7.61-7.57 (m, 1H), 7.50-7.47 (m, 2H), 7.04 (s, 1H), 0.31 (s, 9H); ^13^C NMR (100 MHz, CDCl_3_) δ 191.4, 148.6, 136.7, 133.5, 133.4, 128.9, 128.7, -2.5. HRMS (ESI): m/z calcd. for C_12_H_15_ClOSi [M + Na]^+^ 261.0473, found 261.0477.

**(*Z*)-3-Chloro-3-phenyl-1-(p-tolyl)prop-2-en-1-one^1^ (4a)**

Prepared according to the general procedure using PdCl_2_(PPh_3_)_2_ (2.8 mg, 0.02 equiv.), CuI (1.5 mg, 0.04 equiv.), Et_3_N (33.3 μL, 1.2 equiv.), phenylacetylene (21.7 μL, 0.2 mmol) and 4-methylbenzoyl chloride (34.4 μL, 0.26 mmol), and DCE (0.5 mL) sequentially under N_2_ and then the resulting mixture was stirred at room temperature for 10 min. HOTf (26.5 μL, 1.5 equiv.) was added to the reaction, stirred at room temperature for 4 h. The crude reaction mixture was purified by flash column chromatography using PE/EA = 100/1 as the eluent to give 46.6 mg (87% yield) of **4a** (Z/E = 93/7) as a pale yellow solid; mp: 71-73 ^o^C; ^1^H NMR (400 MHz, CDCl_3_) δ 7.91 (d, *J* = 8.0 Hz, 2H), 7.81-7.73 (m, 2H), 7.45-7.43 (m, 3H), 7.33 (s, 1H), 7.29 (d, *J* = 8.0 Hz, 2H), 2.43 (s, 3H); ^13^C NMR (100 MHz, CDCl_3_) δ 189.6, 144.3, 142.6, 137.3, 135.1, 130.4, 129.4, 128.8, 128.6, 127.1, 121.8, 21.7.

**(*Z*)-3-Chloro-1-(4-methoxyphenyl)-3-phenylprop-2-en-1-one^1^ (4b)**

Prepared according to the general procedure using PdCl_2_(PPh_3_)_2_ (2.8 mg, 0.02 equiv.), CuI (1.5 mg, 0.04 equiv.), Et_3_N (33.3 μL, 1.2 equiv.), phenylacetylene (21.7 μL, 0.2 mmol) and 4-methoxybenzoyl chloride (44.4 mg, 0.26 mmol), and DCE (0.5 mL) sequentially under N_2_ and then the resulting mixture was stirred at room temperature for 10 min. HOTf (26.5 μL, 1.5 equiv.) was added to the reaction, stirred at room temperature for 4 h. The crude reaction mixture was purified by flash column chromatography using PE/EA = 100/1 as the eluent to give 40.5 mg (75% yield) of **4b** (Z/E = 91/9) as a pale yellow oil; ^1^H NMR (400 MHz, CDCl_3_) δ 8.00 (d, *J* = 8.8 Hz, 2H), 7.76-7.74 (m, 2H), 7.45-7.43 (m, 3H), 7.29 (s, 1H), 6.96 (d, *J* = 8.8 Hz, 2H), 3.88 (s, 3H); ^13^C NMR (100 MHz, CDCl_3_) δ 188.7, 163.8, 141.9, 137.3, 131.1, 130.5, 130.3, 128.6, 127.0, 122.0, 113.9, 55.5.

**(*Z*)-3-Chloro-4,4-dimethyl-1-(p-tolyl)pent-2-en-1-one (4c)**

Prepared according to the general procedure using PdCl_2_(PPh_3_)_2_ (2.8 mg, 0.02 equiv.), CuI (1.5 mg, 0.04 equiv.), Et_3_N (33.3 μL, 1.2 equiv.), 3,3-dimethylbut-1-yne (24.6 μL, 0.2 mmol) and 4-methylbenzoyl chloride (34.4 μL, 0.26 mmol), and DCE (0.5 mL) sequentially under N_2_ and then the resulting mixture was stirred at room temperature for 10 min. HOTf (26.5 μL, 1.5 equiv.) was added to the reaction, stirred at room temperature for 4 h. The crude reaction mixture was purified by flash column chromatography using PE/EA = 50/1 as the eluent to give 40.7 mg (92% yield) of **4c** (Z/E > 99/1) as a pale yellow oil; ^1^H NMR (400 MHz, CDCl_3_) δ 7.83 (d, *J* = 8.4 Hz, 2H), 7.27-7.25 (m, 2H), 6.68 (s, 1H), 2.42 (s, 3H), 1.32 (s, 6H); ^13^C NMR (100 MHz, CDCl_3_) δ 191.4, 153.9, 144.2, 134.8, 129.3, 129.0, 119.7, 39.8, 28.7, 21.7. HRMS (ESI): m/z calcd. for C_14_H_17_ClO [M+H]^+^ 237.1041, found 237.1048.

**(*Z*)-3-chloro-4,4-dimethyl-1-(m-tolyl)pent-2-en-1-one (4d)**

Prepared according to the general procedure using PdCl_2_(PPh_3_)_2_ (2.8 mg, 0.02 equiv.), CuI (1.5 mg, 0.04 equiv.), Et_3_N (33.3 μL, 1.2 equiv.), 3,3-dimethylbut-1-yne (24.6 μL, 0.2 mmol) and 3-methylbenzoyl chloride (34.4 μL, 0.26 mmol), and DCE (0.5 mL) sequentially under N_2_ and then the resulting mixture was stirred at room temperature for 10 min. HOTf (26.5 μL, 1.5 equiv.) was added to the reaction, stirred at room temperature for 4 h. The crude reaction mixture was purified by flash column chromatography using PE/EA = 20/1 as the eluent to give 38.8 mg (81% yield) of **4d** (Z/E > 99/1) as a pale yellow oil; ^1^H NMR (400 MHz, CDCl_3_) δ 7.74 (s, 1H), 7.70 (d, *J* = 7.2 Hz, 1H), 7.44-7.29 (m, 2H), 6.71 (s, 1H), 2.41 (s, 3H), 1.32 (s, 9H); ^13^C NMR (100 MHz, CDCl_3_) δ 191.8, 154.4, 138.4, 137.3, 134.0, 129.1, 128.4, 126.1, 119.6, 39.8, 28.6, 21.3. HRMS (ESI): m/z calcd. for C_14_H_17_ClO [M+H]^+^ 237.1041, found 237.1053.

**(*Z*)-3-Chloro-1-(4-methoxyphenyl)-4,4-dimethylpent-2-en-1-one (4e)**

Prepared according to the general procedure using PdCl_2_(PPh_3_)_2_ (2.8 mg, 0.02 equiv.), CuI (1.5 mg, 0.04 equiv.), Et_3_N (33.3 μL, 1.2 equiv.), 3,3-dimethylbut-1-yne (24.6 μL, 0.2 mmol) and 4-methoxybenzoyl chloride (44.4 μL, 0.26 mmol), and DCE (0.5 mL) sequentially under N_2_ and then the resulting mixture was stirred at room temperature for 10 min. HOTf (26.5 μL, 1.5 equiv.) was added to the reaction, stirred at room temperature for 4 h. The crude reaction mixture was purified by flash column chromatography using PE/EA = 30/1 as the eluent to give 31.1 mg (63% yield) of **4e** (Z/E > 99/1) as a pale yellow oil; ^1^H NMR (400 MHz, CDCl_3_) δ 7.91 (d, *J* = 8.8 Hz, 2H), 6.94 (d, *J* = 8.8 Hz, 2H), 6.63 (s, 1H), 3.87 (s, 2H), 1.30 (s, 9H); ^13^C NMR (100 MHz, CDCl_3_) δ 190.6, 163.7, 153.2, 131.3, 130.2, 119.9, 113.8, 55.5, 39.7, 28.7. HRMS (ESI): m/z calcd. for C_14_H_17_ClO_2_ [M + Na]^+^ 275.0809, found 275.0805.

**(*Z*)-3-Chloro-4,4-dimethyl-1-(o-tolyl)pent-2-en-1-one (4f)**

Prepared according to the general procedure using PdCl_2_(PPh_3_)_2_ (2.8 mg, 0.02 equiv.), CuI (1.5 mg, 0.04 equiv.), Et_3_N (33.3 μL, 1.2 equiv.), 3,3-dimethylbut-1-yne (24.6 μL, 0.2 mmol) and 2-methylbenzoyl chloride (33.9 μL, 0.26 mmol), and DCE (0.5 mL) sequentially under N_2_ and then the resulting mixture was stirred at room temperature for 10 min. HOTf (26.5 μL, 1.5 equiv.) was added to the reaction, stirred at room temperature for 4 h. The crude reaction mixture was purified by flash column chromatography using PE/EA = 30/1 as the eluent to give 41 mg (89% yield) of **4f** (Z/E > 99/1) as a pale yellow oil; ^1^H NMR (400 MHz, CDCl_3_) δ 7.57 (d, *J* = 7.2 Hz, 1H), 7.40-7.36 (m, 1H), 7.27-7.24 (m, 2H), 6.61 (s, 1H), 2.53 (s, 3H), 1.29 (s, 9H); ^13^C NMR (100 MHz, CDCl_3_) δ 194.6, 154.8, 138.4, 138.1, 131.7, 131.4, 129.5, 125.6, 121.8, 39.9, 28.6, 20.9. HRMS (ESI): m/z calcd. for C_14_H_17_ClO [M + Na]^+^ 259.0860, found 259.0858.

**(*Z*)-3-Chloro-1-(4-fluorophenyl)-4,4-dimethylpent-2-en-1-one (4g)**

Prepared according to the general procedure using PdCl_2_(PPh_3_)_2_ (2.8 mg, 0.02 equiv.), CuI (1.5 mg, 0.04 equiv.), Et_3_N (33.3 μL, 1.2 equiv.), 3,3-dimethylbut-1-yne (24.6 μL, 0.2 mmol) and 4-fluorobenzoyl chloride (30.7 μL, 0.26 mmol), and DCE (0.5 mL) sequentially under N_2_ and then the resulting mixture was stirred at room temperature for 10 min. HOTf (26.5 μL, 1.5 equiv.) was added to the reaction, stirred at room temperature for 4 h. The crude reaction mixture was purified by flash column chromatography using PE/EA = 20/1 as the eluent to give 42.4 mg (88% yield) of **4g** (Z/E > 99/1) as a pale yellow solid; mp 54-55 ^o^C; ^1^H NMR (400 MHz, CDCl_3_) δ 7.97-7.93 (m, 2H), 7.17-7.12 (m, 2H), 6.65 (s, 1H), 1.31 (s, 9H); ^13^C NMR (100 MHz, CDCl_3_) δ 190.2, 165.9 (d, *J* = 254 Hz), 154.7, 133.7 (d, *J* = 2.8 Hz), 131.5 (d, *J* = 9.4 Hz), 119.3, 115.8 (d, *J* = 22.0 Hz), 39.9, 28.6. HRMS (ESI): m/z calcd. for C_13_H_14_ClFO [M + Na]^+^ 263.0609, found 263.0613.

**(*Z*)-3-Chloro-1-(4-chlorophenyl)-4,4-dimethylpent-2-en-1-one (4h)**

Prepared according to the general procedure using PdCl_2_(PPh_3_)_2_ (2.8 mg, 0.02 equiv.), CuI (1.5 mg, 0.04 equiv.), Et_3_N (33.3 μL, 1.2 equiv.), 3,3-dimethylbut-1-yne (24.6 μL, 0.2 mmol) and 4-chlorobenzoyl chloride (33.2 μL, 0.26 mmol), and DCE (0.5 mL) sequentially under N_2_ and then the resulting mixture was stirred at room temperature for 10 min. HOTf (26.5 μL, 1.5 equiv.) was added to the reaction, stirred at room temperature for 4 h. The crude reaction mixture was purified by flash column chromatography using PE/EA = 20/1 as the eluent to give 40.9 mg (80% yield) of **4h** (Z/E > 99/1) as a pale yellow solid; mp 64-66 ^o^C; ^1^H NMR (400 MHz, CDCl_3_) δ 7.90 (d, *J* = 8.4 Hz, 2H), 7.48 (d, *J* = 8.4 Hz, 2H), 6.70 (s, 1H), 1.36 (s, 9H); ^13^C NMR (100 MHz, CDCl_3_) δ 190.4, 155.2, 139.7, 135.6, 130.2, 128.9, 119.0, 39.9, 28.6. HRMS (ESI): m/z calcd. for C_13_H_14_Cl_2_O [M + Na]^+^ 279.0314, found 279.0327.

**(*Z*)-1-(4-Bromophenyl)-3-chloro-4,4-dimethylpent-2-en-1-one (4i)**

Prepared according to the general procedure using PdCl_2_(PPh_3_)_2_ (2.8 mg, 0.02 equiv.), CuI (1.5 mg, 0.04 equiv.), Et_3_N (33.3 μL, 1.2 equiv.), 3,3-dimethylbut-1-yne (24.6 μL, 0.2 mmol) and 4-bromobenzoyl chloride (35.4 μL, 0.26 mmol), and DCE (0.5 mL) sequentially under N_2_ and then the resulting mixture was stirred at room temperature for 10 min. HOTf (26.5 μL, 1.5 equiv.) was added to the reaction, stirred at room temperature for 4 h. The crude reaction mixture was purified by flash column chromatography using PE/EA = 20/1 as the eluent to give 41 mg (68% yield) of **4i** (Z/E > 99/1) as a pale yellow solid; mp 68-69 ^o^C; ^1^H NMR (400 MHz, CDCl_3_) δ 7.77 (d, *J* = 8.4 Hz, 2H), 7.60 (d, *J* = 8.4 Hz, 2H), 6.65 (s, 1H), 1.31 (s, 9H); ^13^C NMR (100 MHz, CDCl_3_) δ 190.6, 155.3, 136.1, 131.9, 130.3, 128.4, 119.0, 40.0, 28.6. HRMS (ESI): m/z calcd. for C_13_H_14_BrClO [M + Na]^+^ 322.9809, found 322.9795.

**(*Z*)-3-Chloro-1-(3-chlorophenyl)-4,4-dimethylpent-2-en-1-one (4j)**

Prepared according to the general procedure using PdCl_2_(PPh_3_)_2_ (2.8 mg, 0.02 equiv.), CuI (1.5 mg, 0.04 equiv.), Et_3_N (33.3 μL, 1.2 equiv.), 3,3-dimethylbut-1-yne (24.6 μL, 0.2 mmol) and 3-chlorobenzoyl chloride (33.3 μL, 0.26 mmol), and DCE (0.5 mL) sequentially under N_2_ and then the resulting mixture was stirred at room temperature for 10 min. HOTf (26.5 μL, 1.5 equiv.) was added to the reaction, stirred at room temperature for 4 h. The crude reaction mixture was purified by flash column chromatography using PE/EA = 20/1 as the eluent to give 42.4 mg (82% yield) of **4j** (Z/E > 99/1) as a pale yellow solid; mp 55-56 ^o^C; ^1^H NMR (400 MHz, CDCl_3_) δ 7.88-7.87 (m, 1H), 7.77 (d, *J* = 8.0 Hz, 2H), 7.55-7.53 (m, 2H), 7.43-7.39 (m, 1H), 6.68 (s, 1H), 1.31 (s, 9H); ^13^C NMR (100 MHz, CDCl_3_) δ 190.2, 167.1, 164.6, 154.7, 131.5, 131.4, 119.3, 115.9, 115.6, 39.9, 28.6. HRMS (ESI): m/z calcd. for C_13_H_14_Cl_2_O [M + Na]^+^ 279.0314, found 279.0323.

**(*Z*)-1-(2-Bromophenyl)-3-chloro-4,4-dimethylpent-2-en-1-one (4k)**

Prepared according to the general procedure using PdCl_2_(PPh_3_)_2_ (2.8 mg, 0.02 equiv.), CuI (1.5 mg, 0.04 equiv.), Et_3_N (33.3 μL, 1.2 equiv.), 3,3-dimethylbut-1-yne (24.6 μL, 0.2 mmol) and 2-bromobenzoyl chloride (34.1 μL, 0.26 mmol), and DCE (0.5 mL) sequentially under N_2_ and then the resulting mixture was stirred at room temperature for 10 min. HOTf (26.5 μL, 1.5 equiv.) was added to the reaction, stirred at room temperature for 4 h. The crude reaction mixture was purified by flash column chromatography using PE/EA = 20/1 as the eluent to give 43.5 mg (72% yield) of **4k** (Z/E > 99/1) as a pale yellow solid; mp 64-66 ^o^C; ^1^H NMR (400 MHz, CDCl_3_) δ 7.59-7.61 (m, 1H), 7.49-7.46 (m, 1H), 7.40-7.36 (m, 1H), 7.33-7.29 (m, 1H), 6.68 (s, 1H), 1.27 (s, 9H); ^13^C NMR (100 MHz, CDCl_3_) δ 192.5, 157.4, 141.6, 133.5, 131.8, 129.9, 127.5, 121.2, 119.5, 40.3, 28.5. HRMS (ESI): m/z calcd. for C_13_H_14_BrClO [M + Na]^+^ 322.9809, found 322.9804.

**(Z)-4-(3-chloro-3-phenylacryloyl)benzonitrile (4l)**

Prepared according to the general procedure using PdCl_2_(PPh_3_)_2_ (2.8 mg, 0.02 equiv.), CuI (1.5 mg, 0.04 equiv.), Et_3_N (33.3 μL, 1.2 equiv.), 3,3-dimethylbut-1-yne (24.6 μL, 0.2 mmol) and 4-cyanobenzoyl chloride (43.1 mg, 0.26 mmol), and DCE (0.5 mL) sequentially under N_2_ and then the resulting mixture was stirred at room temperature for 10 min. HOTf (26.5 μL, 1.5 equiv.) was added to the reaction, stirred at room temperature for 4 h. The crude reaction mixture was purified by purified by prep-TLC using PE/EA = 10/1 as the eluent to give 35.9 mg (82% yield) of **4l** (Z/E > 99/1) as a yellow solid; m. p. 108.8 ^o^C–110.6 ^o^C; ^1^H NMR (500 MHz, CDCl_3_) δ 8.08 (d, *J* = 8.4 Hz, 2H), 7.84–7.73 (m, 4H), 7.54–7.44 (m, 3H), 7.33 (s, 1H); ^13^C NMR (126 MHz, CDCl_3_) δ 188.3, 145.8, 141.0, 136.9, 132.6, 131.3, 129.0, 128.8, 127.3, 120.2, 117.9, 116.4.

**(Z)-3-chloro-3-phenyl-1-(4-(trifluoromethyl)phenyl)prop-2-en-1-one (4m)**

Prepared according to the general procedure using PdCl_2_(PPh_3_)_2_ (2.8 mg, 0.02 equiv.), CuI (1.5 mg, 0.04 equiv.), Et_3_N (33.3 μL, 1.2 equiv.), 3,3-dimethylbut-1-yne (24.6 μL, 0.2 mmol) and 4-(trifluoromethyl)benzoyl chloride (54.2 mg, 0.26 mmol), and DCE (0.5 mL) sequentially under N_2_ and then the resulting mixture was stirred at room temperature for 10 min. HOTf (26.5 μL, 1.5 equiv.) was added to the reaction, stirred at room temperature for 4 h. The crude reaction mixture was purified by purified by prep-TLC using PE/EA = 10/1 as the eluent to give 37.4 mg (70% yield) of **4m** (Z/E > 99/1) as a pale yellow solid; m.p. 80.7 ^o^C–81.7 ^o^C; ^1^H NMR (500 MHz, CDCl_3_) δ 8.09 (d, *J* = 8.0 Hz, 2H), 7.83–7.68 (m, 4H), 7.53–7.40 (m, 3H), 7.35 (s, 1H); ^13^C NMR (126 MHz, CDCl_3_) δ 188.7, 145.1, 140.6,137.1, 134.5 (q, *J* = 32.8 Hz), 131.0, 128.9, 128.8, 127.3, 125.8 (q, *J* = 3.8 Hz), 123.6 (q, *J* = 273.4 Hz), 120.6.

**(*Z*)-3-Chloro-1-cyclohexyl-4,4-dimethylpent-2-en-1-one (4n)**

Prepared according to the general procedure using PdCl_2_(PPh_3_)_2_ (2.8 mg, 0.02 equiv.), CuI (1.5 mg, 0.04 equiv.), Et_3_N (33.3 μL, 1.2 equiv.), 3,3-dimethylbut-1-yne (24.6 μL, 0.2 mmol) and cyclohexanecarbonyl chloride (34.8 μL, 0.26 mmol), and DCE (0.5 mL) sequentially under N_2_ and then the resulting mixture was stirred at room temperature for 10 min. HOTf (26.5 μL, 1.5 equiv.) was added to the reaction, stirred at room temperature for 4 h. The crude reaction mixture was purified by flash column chromatography using PE/EA = 20/1 as the eluent to give 35.9 mg (82% yield) of **4n** (Z/E > 99/1) as a colorless oil; ^1^H NMR (400 MHz, CDCl_3_) δ 6.31 (s, 1H), 2.56-2.50 (m, 1H), 1.87-1.75 (m, 6H), 1.43-1.25 (m, 4H), 1.23 (s, 9H); ^13^C NMR (100 MHz, CDCl_3_) δ 203.1, 154.6, 120.3, 51.3, 40.0, 28.7, 28.3, 25.8, 25.6. HRMS (ESI): m/z calcd. for C_13_H_21_ClO [M + Na]^+^ 251.1173, found 251.1174.

**(*Z*)-3-Chloro-4,4-dimethyl-1-(thiophen-2-yl)pent-2-en-1-one (4o)**

Prepared according to the general procedure using PdCl_2_(PPh_3_)_2_ (2.8 mg, 0.02 equiv.), CuI (1.5 mg, 0.04 equiv.), Et_3_N (33.3 μL, 1.2 equiv.), 3,3-dimethylbut-1-yne (24.6 μL, 0.2 mmol) and thiophene-2-carbonyl chloride (27.8 μL, 0.26 mmol), and DCE (0.5 mL) sequentially under N_2_ and then the resulting mixture was stirred at room temperature for 10 min. HOTf (26.5 μL, 1.5 equiv.) was added to the reaction, stirred at room temperature for 4 h. The crude reaction mixture was purified by flash column chromatography using PE/EA = 20/1 as the eluent to give 34.3 mg (77% yield) of **4o** (Z/E > 99/1) as a pale yellow oil; ^1^H NMR (400 MHz, CDCl_3_) δ 7.72-7.58 (m, 2H), 7.15-7.13 (m, 1H), 6.76 (s, 1H), 1.30 (s, 9H); ^13^C NMR (100 MHz, CDCl_3_) δ 182.8, 156.0, 145.0, 134.1, 132.4, 128.1, 118.4, 40.1, 28.6. HRMS (ESI): m/z calcd. for C_11_H_13_ClOS [M + Na]^+^ 251.0268, found 251.0274.

**(*Z*)-3-Bromo-4,4-dimethyl-1-phenylpent-2-en-1-one (6)**

Prepared according to the general procedure using PdCl_2_(PPh_3_)_2_ (2.8 mg, 0.02 equiv.), CuI (1.5 mg, 0.04 equiv.), Et_3_N (33.3 μL, 1.2 equiv.), 3,3-dimethylbut-1-yne (24.6 μL, 0.2 mmol) and benzoyl bromide (30.6 μL, 0.26 mmol), and DCE (0.5 mL) sequentially under N_2_ and then the resulting mixture was stirred at room temperature for 10 min. HOTf (26.5 μL, 1.5 equiv.) was added to the reaction, stirred at room temperature for 4 h. The crude reaction mixture was purified by flash column chromatography using PE/EA = 20/1 as the eluent to give 39.2 mg (70% yield) of **6** (Z/E > 99/1) as a pale yellow solid; mp 82-84 ^o^C; ^1^H NMR (400 MHz, CDCl_3_) δ 7.92 (d, J = 7.2 Hz, 2H), 7.60-7.56 (m, 1H), 7.50-7.46 (m, 2H), 6.91 (s, 1H), 1.31 (s, 9H); ^13^C NMR (100 MHz, CDCl_3_) δ 192.6, 147.5, 136.6, 133.4, 129.0, 128.6, 123.2, 40.5, 29.3.

## V. References

1 P. Gandeepan, K. Parthasarathy, T.-H. Su and C.-H. Cheng, Iron-Catalyzed Synthesis of β-Chlorovinyl and α,β‐Alkynyl Ketones from Terminal and Silylated Alkynes with Acid Chlorides. *Adv. Synth. Catal.,* **2012**, *354*, 457.

2 B. Wang, S. Wang, P. Li and L. Wang, Iron-Catalyzed Regio- and Stereoselective Addition of Acid Chlorides to Alkynes. *Chem. Commun.,* **2010**, *46*, 5891.

## VI. ^1^H and ^13^C NMR Spectra

**3a**

**3a**

**3b**

**3b**

**3c**

**3c**

**3d**

**3d**

**3e**

**3e**

**3f**

**3f**

**3g**

**3g**

**3h**

**3h**

**3i**

**3i**

**3j**

**3j**

**3k**

**3k**

**3l**

**3l**

**3mmm**

**3mmm**

**4a**

**4a**

**4b**

**4b**

**4c**

**4c**

**4d**

**4d**

**4e**

**4e**

**4f**

**4f**

**4g**

**4g**

**4h**

**4h**

**4i**

**4i**

**4j**

**4j**

**4k**

**4k**

**4l**

**4l**

**4m**

**4m**

**4n**

**4n**

**4o**

**4o**

**6**

**6**
